# Multi-Omics Analysis Reveals Aberrant Gut-Metabolome-Immune Network in Schizophrenia

**DOI:** 10.3389/fimmu.2022.812293

**Published:** 2022-03-03

**Authors:** Yajuan Fan, Yuan Gao, Qingyan Ma, Zai Yang, Binbin Zhao, Xiaoyan He, Jian Yang, Bin Yan, Fengjie Gao, Li Qian, Wei Wang, Feng Zhu, Xiancang Ma

**Affiliations:** ^1^ Department of Psychiatry, The First Afffliated Hospital of Xi’an Jiaotong University, Xi’an, China; ^2^ Center for Brain Science, The First Affiliated Hospital of Xi’an Jiaotong University, Xi’an, China; ^3^ Clinical Research Center for Psychiatric Medicine of Shaanxi Province, The First Affiliated Hospital of Xi’an Jiaotong University, Xi’an, China; ^4^ Clinical Research Center, The First Affiliated Hospital of Xi’an Jiaotong University, Xi’an, China; ^5^ Center for Translational Medicine, The First Affiliated Hospital of Xi’an Jiaotong University, Xi’an, China

**Keywords:** schizophrenia, gut microbiota, metabolism, cytokines, metagenomics

## Abstract

Schizophrenia (SCZ) is associated with several immune dysfunctions, including elevated levels of pro-inflammatory cytokines. Microorganisms and their metabolites have been found to regulate the immune system, and that intestinal microbiota is significantly disturbed in schizophrenic patients. To systematically investigate aberrant gut-metabolome-immune network in schizophrenia, we performed an integrative analysis of intestinal microbiota, serum metabolome, and serum inflammatory cytokines in 63 SCZ patients and 57 healthy controls using a multi-omics strategy. Eighteen differentially abundant metabolite clusters were altered in patients displayed higher cytokine levels, with a significant increase in pro-inflammatory metabolites and a significant decrease in anti-inflammatory metabolites (such as oleic acid and linolenic acid). The bacterial co-abundance groups in the gut displayed more numerous and stronger correlations with circulating metabolites than with cytokines. By integrating these data, we identified that certain bacteria might affect inflammatory cytokines by modulating host metabolites, such as amino acids and fatty acids. A random forest model was constructed based on omics data, and seven serum metabolites significantly associated with cytokines and α-diversity of intestinal microbiota were able to accurately distinguish the cases from the controls with an area under the receiver operating characteristic curve of 0.99. Our results indicated aberrant gut-metabolome-immune network in SCZ and gut microbiota may influence immune responses by regulating host metabolic processes. These findings suggest a mechanism by which microbial-derived metabolites regulated inflammatory cytokines and insights into the diagnosis and treatment of mental disorders from the microbial-immune system in the future.

## Introduction

Cumulative evidence suggests that patients with schizophrenia (SCZ) exhibit a state of immune activation with significantly elevated pro-inflammatory cytokines, such as tumor necrosis factor-α (TNF-α), interleukin (IL)-1β, and IL-6 (1, 2), suggesting that inflammation is a possible risk factor for inducing schizophrenia and exacerbating its symptoms (3, 4). Gut microbiota plays a key role in the early development of the neuroimmune system and is crucial for myelination, synaptic pruning and neuronal remodeling (5). Changes in intestinal microbiota characteristics early in life may lead to immune disorders (6). Recent studies have shown that SCZ patients have altered microbiota α-diversity index and marked disturbances of gut microbial composition and function, including short-chain fatty acid synthesis, amino acid metabolism, and neurotransmitter synthesis/degradation (7–9). Moreover, the microbiota can shape the intestinal microecosystem and influence host physiological processes by producing metabolites that are involved in signaling and immune system regulation (10–12). Wilmanski et al. found that 40 plasma metabolites (13 of the 40 of microbial origin) can explain 45% of the variance in α-diversity, demonstrating a strong association between metabolic output and gut microbial structure (13). So we hypothesized that metabolites play a very important role in regulating the relationship between gut microbes and the immune system in SCZ.

Metabolomic analyses of serum and plasma from SCZ subjects have revealed marked differences compared to healthy controls (HCs), with many dysregulated compounds having microbial origin (8, 14). Notably, amino acid transport and degradation differ between SCZ patients and HCs (15–17). These processes can be regulated by intestinal microbiota and implicated in SCZ pathophysiology because amino acids serve as precursors for many potent neuroactive molecules, such as classic neurotransmitters (18). Some metabolites with neuroprotective and anti-oxidantive inflammatory effects, such as arachidonic acid, oleic acid, and alpha-linolenic acid, are also significantly reduced in SCZ (19, 20). These results suggest that multiple biologically abnormal pathways are involved in SCZ and that no single biological etiology may be responsible for all cases (21). However, most of the current researches focus on understanding the taxonomic composition of the gut microbiota, metabolic disturbance, and immune abnormalities in these patients using monomic approach. Therefore, studying the gut microbiota and metabolite composition simultaneously in patients with SCZ may be critical to our understanding of immune activation of SCZ.

Recent advancements in multi-omics technologies have enabled system-level analysis to identify biomarkers reflecting the states of human health and disease (22). A number of studies have combined microbial-omics and metabonomics to describe the disease-specific structure of the intestinal microbiota and metabolic patterns and their interactions (23–25). A better understanding of the mechanistic roles of the gut microbiota in the regulation of host metabolic and immunological functions will help us understand the biological mechanisms of SCZ. In the present study, we analyzed the gut microbial characteristics of SCZ patients and HCs using shotgun metagenomic sequencing. Additionally, we used untargeted liquid chromatography-mass spectrometry (LC-MS) and bead-based multiplex cytokine assay to analyze the metabolic profiles and cytokines in the subjects’ serum. Based on these analyses, we identified specific gut microbiota and serum metabolite profiles associated with cytokines, and established associations, particularly between bacterial co-abundance groups (CAGs) and serum metabotypes. We suggest a mechanism of action involving the production of microbial metabolites that regulate inflammatory cytokines.

## Methods

### Characteristics of the Study Population

From March 2016 to August 2017, 63 individuals with a primary diagnosis of SCZ, according to the Diagnostic and Statistical Manual of Mental Disorders, were enrolled in the study. All patients were hospitalized at the First Affiliated Hospital of Xi’an Jiaotong University, with a duration of illness of less than 6 years and taking antipsychotics for no more than 1 week in the 6 months before enrollment. During the same period, 57 HCs from the same area were recruited. All subjects were excluded if they had infectious diseases, autoimmune disorders, gastrointestinal diseases, other severe or unstable medical illness, comorbidities with other psychiatric disorders (including alcohol and substance use disorders), or were administered with antibiotics for more than 3 days in the previous 3 months. All clinical information was collected according to standard procedures, including age, sex, body mass index (BMI), education, course of illness, dietary habits, and bowel habits. All subjects were informed of the study details and signed a written consent form. The Ethics Committee of the First Affiliated Hospital of Xi’an Jiaotong University reviewed and approved the research protocol (XJTY1AF2015LSL-079).

Peripheral venous blood (4–5 mL) was drawn after overnight fasting. After allowing to stand, the serum was centrifuged at 2000 r/min for 10 min. Subsequently, serum samples were extracted and transferred into Eppendorf tubes, which were subsequently stored at −80°C until untargeted LC-MS analysis or multiplex cytokine assay. Stool samples were collected by the researchers according to the standard operating procedures. Newly collected fecal samples from each subject were immediately transported to the laboratory and frozen at −80°C.

In addition, we also included a small validation cohort, including 23 SCZ patients and 23 HCs who met the same inclusion and exclusion criteria as the above cohort. Serum bile acid levels of all subjects in the cohort were measured by LC-MS.

### Untargeted LC-MS/MS Analysis

Fifty microliters of the sample was transferred to an EP tube. After the addition of 150 μL of methanol (containing 1 μg/mL internal standard of mass-spec), the samples were vortexed for 30 s, sonicated for 10 min in an ice-water bath, and incubated for 1 h at -40°C to precipitate proteins. The sample was then centrifuged at 12000 rpm for 15 min at 4°C. The resulting supernatant was transferred to a fresh glass vial for further analysis. The quality control sample was prepared by mixing an equal aliquot of the supernatant from all samples.

LC-MS/MS analyses were performed using a UHPLC system (Vanquish, Thermo Fisher Scientific) with a UPLC BEH Amide column (2.1 mm × 100 mm, 1.7 μm) coupled to a Q Exactive HFX mass spectrometer (Orbitrap MS, Thermo). The mobile phase consisted of 25 mmol/L ammonium acetate and 25 ammonia hydroxide in water (pH = 9.75) (A) and acetonitrile (B). The analysis was carried with elution gradient as follows: 0~0.5 min, 95% B; 0.5~7.0 min, 95%~65% B; 7.0~8.0 min, 65%~40% B; 8.0~9.0 min, 40% B; 9.0~9.1 min, 40%~95% B; 9.1~12.0 min, 95% B. The column temperature was maintained at 35°C. The auto-sampler temperature was 4°C, and the injection volume was 3 μL.

The QE HFX mass spectrometer was used for its ability to acquire MS/MS spectra in information-dependent acquisition mode in the control of the acquisition software (Xcalibur, Thermo). In this mode, the acquisition software continuously evaluates the full-scan MS spectrum. The ESI source conditions were set as following: sheath gas flow rate as 50 Arb, Aux gas flow rate as 10 Arb, capillary temperature 320°C, full MS resolution as 60000, MS/MS resolution as 7500, collision energy as 10/30/60 in NCE mode, and spray Voltage as 3.5 kV (positive) or -3.2 kV (negative).

The raw data were converted to the mzXML format using ProteoWizard and processed with an in-house program, which was developed using R and based on XCMS, for peak detection, extraction, alignment, and integration. The online HMDB database (http://www.hmdb.ca) (version: 4.0) and an in-house MS2 database (BiotreeDB) were used for metabolite annotation. The cut-off for annotation was set at 0.3.

### Metabolomics Data Analysis

After normalization and integration using support vector regression, the processed data were uploaded into SIMCA-P software 14.1 (Umetrics, Umeå, Sweden) and MetaboAnalyst software (version 3.0, www.metaboanalyst.ca) for further analysis. The variable importance in the projection (VIP) value of each variable in the orthogonal partial least squares discriminant analysis (OPLS-DA) model was calculated to show its contribution to classiffcation. Metabolites with VIP values > 1 were further subjected to Student’s t-test at the univariate level to measure the signiffcance of each metabolite. The Benjamini-Hochberg procedure was used for multiple testing and the critical false discovery rate (FDR) set to 0.05.

Clusters of co-abundant serum metabolites were identified using the weighted gene co-expression network analysis (WGCNA) (26). Signed, weighted metabolite co-abundance correlation networks were calculated across all enrolled individuals. The soft threshold β = 13 of serum metabolite correlation was selected by scale-free topology criterion. Dynamic hybrid tree cutting algorithm with deepSplit of 4 was used to identify clusters.

### Shotgun Metagenomic Sequencing and Metagenome-Wide Association Study

Shotgun metagenomic sequencing and alignment methods were based on our previously published literature (9). Shannon index and partial least squares discriminant analysis were used to calculate α-diversity (within-sample diversity) and β-diversity (between-sample diversity) were calculated depending on the metagenomic operational taxonomic unit (mOTU) proffle, respectively (9, 27). Wilcoxon rank-sum test was used to compare the relative abundance of each mOTU between patients and controls. The correlations between mOTUs and diagnosis were evaluated by the semi-partial Kendall correlation tests, and age, sex, and body mass index (BMI) were adjusted (R 3.5.1, ppcor package). Subsequently, the mOTUs were clustered using the Ward clustering algorithm *via* the R package WGCNA. The mOTU co-occurrence and CAG network were visualized using Cytoscape 3.4.0. All metagenomic raw data have been submitted to GSA (accession number CRA004662).

### Multiplex Cytokine Measurements

Multiple human cytokines in serum were simultaneously analyzed using a bead-based multiplex cytokine assay (Luminex, USA). Levels of IL-1β, IL-4, IL-6, C-C motif chemokine ligand 2 (CCL2), interferon (IFN)-γ, and TNF-α were measured using a human Premixed Multi-Analyte Kit (LXSAHM-04, R&D Systems, USA), according to the manufacturer’s instructions. The assay for each cytokine was performed using the Luminex200 multiplex assay detection system (Luminex, USA).

### Statistical Analyses

Statistical analyses were performed using the Statistical Package for the Social Sciences (SPSS) version 23.0 (Armonk, NY: International Business Machines Corp.). Continuous, non-normally distributed variables are presented as median and interquartile range and then were analyzed using the Wilcoxon rank-sum test. Categorical variables between groups were compared using the χ2 test. The difference was statistically significant (*P* < 0.05)

Spearman correlations between CAGs, metabotypes, and cytokines were calculated using R 3.5.1, and the Benjamini-Hochberg method was used to control FDR. The visual presentations of multiple omics correlations were performed using R (ggcor and ggplot2 packages). For metabolite and cytokine associated with the same mOTUs, spearman correlations was first used to check whether the metabolite was also associated with the cytokine. Next, directional mediation analysis was performed to infer the role of the microbiota in regulating cytokines through metabolites (mediation package).

The cohort samples were randomly divided into a training set (70%) and a test set (30%). Five-fold cross-validation was performed ten times on a random forest model using mOTU and metabolite abundance proffles within the training set. We selected an optimal set of variables with the lowest cross-validation error. The predictive model was constructed using the most important variables, and further applied for receiver operating characteristic (ROC) analysis in training set and test set (pROC package), respectively.

## Results

### Subject Characteristics

A total of 63 individuals with SCZ and 57 HCs were enrolled in the study. A flowchart of the study design is presented in **Figure S1**. The demographic information and inflammatory cytokines of the 120 subjects are summarized in **Table S1**. All cytokines tested showed increasing trends in the SCZ group, and the serum levels of CCL2 and IFN-γ were significantly higher than the HC group. In addition, there were no significant differences in dietary habits and bowel habits between the two groups.

### Differential Metabotypes Perturbed in SCZ

**Figure 1:**
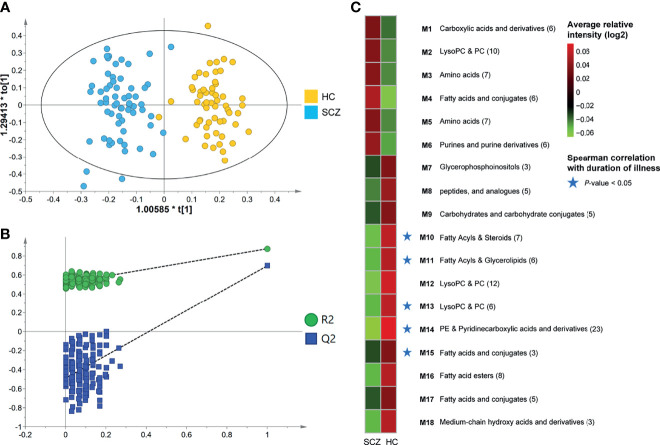
Identification of differential metabotypes associated with schizophrenia. **(A)** The orthogonal partial least squares discriminant analysis (OPLS-DA) model was used to discriminate between 63 SCZ subjects (blue boxes) and 57 HCs (yellow diamonds). **(B)** Permutation test showing the original R2 and Q2 values (top right) as significantly higher than corresponding permuted values (bottom left), demonstrating the OPLS-DA model’s robustness. **(C)** Average relative intensities of the 18 co-abundance metabotypes (clusters) between the two groups. Red and green represented relatively high and low densities, respectively. The asterisk represented a significant negative correlation between the metabotype and the duration of illness (*P*-value < 0.05). .

To gain a deeper understanding of the composition of serum metabolome features in SCZ patients, we used untargeted LC-MS to detect metabolome profiles in fasting serum samples. In the obtained raw data, 8488 features were yielded by the two ion modes, and nearly 19% of the features were significantly altered (OPLS-DA VIP > 1.0, FDR < 0.05). The OPLS-DA score plot showed a significant discrimination between the two groups (**Figure 1A**), suggesting that metabolic disturbance under pathological conditions was evident in the patients, and the validation plot confirmed the validity of the OPLS-DA model (**Figure 1B**). We further found that global metabolite profiles were not greatly influenced by age, sex, or BMI (**Figures S2A–C**).

Through annotation and retrieval, 133 host-derived or bacteria-derived metabolite features were significantly different in SCZ, and the majority (89 [67%]) were significantly depleted in SCZ (**Table S2**). Compared with HCs, SCZ exhibited an increase in oxidative stress metabolites, such as asymmetric dimethylarginine, hydroxyisocaproic acid, and 1-methylguanosine, and a decrease in anti-inflammatory and neuroprotective metabolites, such as oleic acid, arachidonic acid, alpha-linolenic acid, resolvin D2, and 1-methylnicotinamide (MNA). Furthermore, we found that 13 of these metabolites were reported in the literature and the Human Metabolome Database. Alterations of eight metabolites, including oleic acid, taurine, arachidonic acid, perillic acid, L-glutamic acid, L-phenylalanine, L-serine, and allantoin, were consistent with the results reported in the literature, and the remaining five, including tryptophan, betaine, myo-inositol, LysoPC[18:2(9Z,12Z)], and pyrrolidonecarboxylic acid, were relatively controversial. To characterize the metabolic disturbance of SCZ in as much detail as possible and discover new potential biomarkers, we further investigated the significant correlations between the newly discovered metabolites and 13 previously reported metabolites (**Figure S2D**) and found a panel of 12 metabolites derived from fatty acids that have anti-inflammatory and antioxidant effects (Panel 1 in **Figure S2D**). All metabolites in that panel were downregulated in SCZ patients and displayed significant intercorrelations in the whole cohort.

To further explore the biological patterns underlying the 133 differentially abundant (DA) metabolites, we divided the DA metabolites into 18 co-abundance clusters across all subjects using the co-expression network analysis (**Figure 1C** and **Table S3**), and five metabolites were not clustered into any cluster. Metabolites co-clustered by this method will therefore tend to co-vary, and each cluster is assigned to a metabotype *via* their most representative metabolites, that is, matabotype (M) 1-18 (**Table S3**). The largest cluster contained 23 metabolite features, all of which were elevated among HCs, of which 11 features were annotated as phosphatidylethanolamine (PE). M 15, 16, and 17 were also decreased in SCZ patients and were derived from fatty acids and conjugates, including arachidonic acid, oleic acid, alpha-linolenic acid, and eicosadienoic acid (**Figure S3**), some of which play neuroprotective and anti-oxidative inflammatory roles (19, 20). Other clusters of interest included M3 and M5, which were consistently elevated in the SCZ group and contained a variety of amino acid metabolites, including tryptophan, L-serine, and asymmetric dimethylarginine (**Figure S4**). Notably, five metabotypes (M10, 11, 13, 14, 15), which decreased in SCZ patients, were negatively correlated with the duration of illness (**Figure 1C**). The metabolites in these metabotypes mainly included fatty acids, pyridinecarboxylic acids and derivatives.

### Co-Abundance Group Alterations in SCZ Microbial Community Composition

Considering that various serum metabolites in SCZ may come from gut bacteria (28, 29), we investigated gut microbial alterations in SCZ patients. Gut microbiota was profiled by sequencing the fecal genome, and 327 mOTUs were present in more than 5% of the samples (**Table S5**). The gut microbiota of schizophrenic patients showed greater α-diversity at the mOTU level (**Figure S5A**). Based on the β-diversity analysis, we also found that the gut microbiota composition of SCZ patients was significantly different from that of HCs, and these effects were not evidently affected by age, sex, or BMI (**Figures S5C-F**). At the phylum level, the SCZ group was characterized by higher Actinobacteria and Firmicutes levels and significantly lower Bacteroidetes levels (**Figure S5B** and **Table S4**).

A total of 50 mOTUs showed differential relative abundance between the two groups, and 47 mOTUs were still significant after adjusting for age, sex, and BMI. Thirty-nine mOTUs were elevated in SCZ relative to HCs, including two Bacteroides, two Clostridium taxa, two Coprococcus taxa, two Dorea taxa, two Parabacteroides taxa, and two Ruminococcus taxa. Eleven mOTUs were significantly lower in the SCZ group than in the HC group, including three Prevotella taxa, two Streptococcus taxa, and other taxa (**Table S5**). We then found that SCZ-enriched mOTUs were more interconnected than HC-enriched mOTUs by constructing a co-occurrence correlation network (**Figure S6**). Moreover, mOTUs from the same genus had a stronger positive correlation with each other.

**Figure 2:**
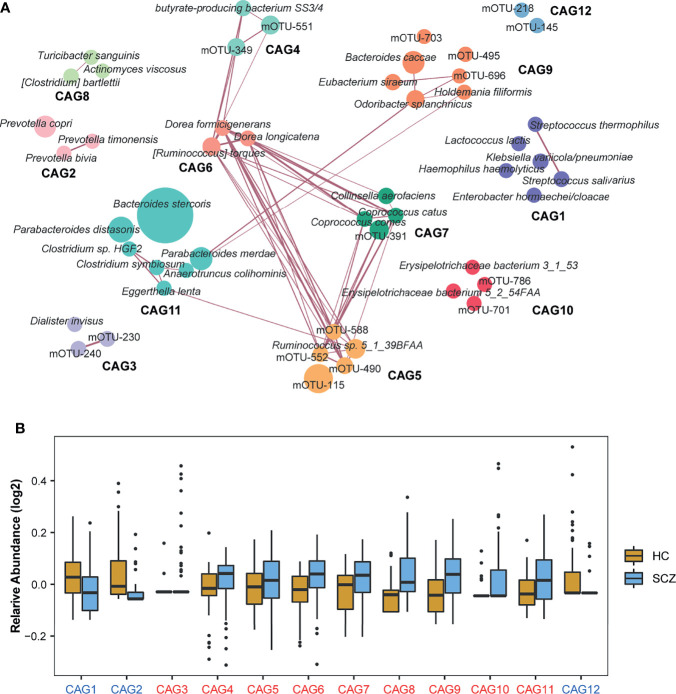
Identification of the important co-abundance groups (CAGs) that were strikingly different between two groups. **(A)** Metagenomic operational taxonomic unit (mOTU)-level network diagram showing the enrichments of mOTUs in the different groups based on significantly changed CAGs. Node size indicated the mean abundance of each mOTU. Lines between nodes represented correlations, with line width indicating correlation magnitude, purple representing positive correlation. Only lines corresponding to correlations with magnitudes greater than 0.4 were drawn. Different colors were used to distinguish CAGs. **(B)** Relative abundances of 12 CAGs with significantly differences identified by the Wilcoxon rank-sum test between the two groups (*P*-value < 0.05). The names of the CAGs comprising the schizophrenia-enriched and healthy control-enriched CAGs were highlighted in red and blue, respectively.

As bacteria work as functional groups termed “guilds” in the gut ecosystem (30), we then clustered the 50 mOTUs into 12 CAGs by constructing a co-abundance network (**Figure 2A**). Each CAG contained 2-7 mOTUs. Of these, CAG1, CAG2, and CAG12 decreased significantly in SCZ patients compared to HCs (**Figure 2B**). Interestingly, all bacteria in CAG2 that were from the Prevotellaceae family, such as *Prevotella bivia*, *Prevotella timonensis*, and *Prevotella copri*, have been reported as probiotics and are related to high-fiber, non-Western diet (31). CAG5, CAG6, and CAG7, whose relative abundances increased in the SCZ group, were closely related to each other. Of the mOTUs in these CAGs, 67% belonged to the Ruminococcaceae and Lachnospiraceae families, members of which may produce short-chain fatty acids (SCFAs) (32). Moreover, butyrate-producing bacterium SS3/4 also showed a significant positive correlation with Lachnospiraceae, which indicated that microbial-derived SCFAs, especially butyric acid, may play an important role in the pathogenesis of SCZ. Through Spearman correlation analysis, we found that CAG1, enriched in the HCs, was negatively correlated with the duration of illness, whereas CAG6 and CAG8, enriched in SCZ, were positively correlated with the duration of illness, which suggested that intestinal bacteria constantly changed with the disease state, and also provided candidate bacteria for constructing disease prediction models and studying endophenotypes in SCZ (**Figure S5G**). The correlations were still significant after adjusting for age, sex, and BMI.

### Multi-Omics Analysis Reveals the Association Among the Gut Microbiota, Serum Metabolites, and Inflammatory Cytokines in SCZ

**Figure 3:**
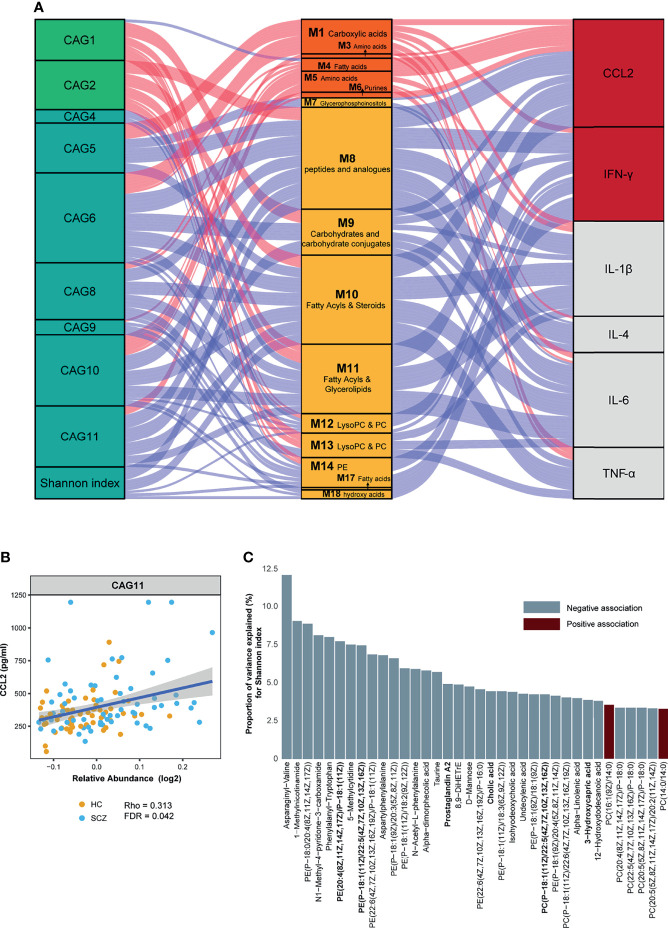
Interrelationship between gut microbiota composition, host metabolic profile, and inflammatory cytokines. **(A)** Visualization of the correlation network according to Spearman correlation analysis between the gut microbiota of significant co-abundance groups (CAGs) and the inflammatory cytokines was mediated by serum metabolites. Red connections indicated positive correlations (Spearman correlation test, FDR < 0.05), whereas blue connections showed correlations that were negative (Spearman correlation test, FDR < 0.05). All correlations were adjusted for age, sex, body mass index, and diet. In the gut microbiota column, the green stratum represented CAGs that were highly enriched in the healthy control (HC) group, and the stratum colored in blue was increased in the schizophrenia (SCZ) group. In the metabolomics column, the orange stratum represented HC-enriched metabotypes, and the claybank stratum represented SCZ-enriched metabotypes. In the cytokines column, the red stratum represented elevated inflammatory cytokines in the SCZ group, and the gray represented no difference between the two groups. **(B)** Scatter plot representing the association between C–C motif chemokine ligand 2 and CAG11 in all subjects. **(C)** The percentage of variance in Shannon index that was explained by each serum metabolite that was significantly associated with Shannon index (*P*-value < 0.05). For each metabolite, red bars correspond to a positive β-coefficient, whereas blue bars correspond to a negative β-coefficient. Significance was assessed using ordinary least squares regression.

Gut microbes and serum metabolites could co-vary as a result of mutual interactions or bidirectional modulation. To determine such associations, we assessed a large-scale association between the metabotypes and CAGs across all subjects. Given an FDR of 5%, nine CAGs and Shannon index were significantly correlated with 15 metabotypes, presenting 67 marked associations (**Figure 3A** and **Table S6**). CAG1 and CAG2, enriched in the HC group, were also positively correlated with HC-enriched metabotypes, such as fatty acyls, LysoPC/PC, and carbohydrate conjugates, but negatively correlated with “SCZ-enriched metabotypes”, such as fatty acids and conjugates. The CAGs enriched in SCZ showed significant associations with metabotypes in the opposite direction to the above results. Among them, CAG5 and CAG6 were also significantly correlated with metabolites such as fatty acids and phospholipids. Using linear regression, 36 of the 133 DA metabolites were significantly associated with Shannon diversity (**Figure 3C**). The metabolites involved in amino acids and pyridinecarboxylic acids, such as asparaginyl-valine and MNA, displayed the strongest associations. The most abundant metabolites associated with the Shannon index were glycerophospholipids.

**Figure 4:**
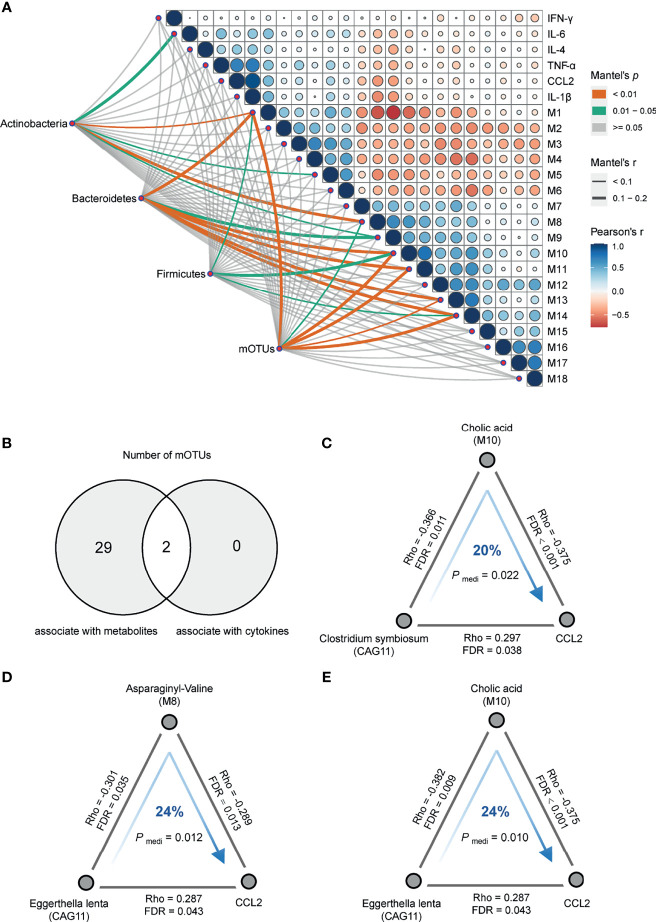
Correlation analysis of multi-omics. **(A)** Pairwise comparisons of metabotypes and cytokines were shown, with a color gradient denoting Pearson’s correlation coefficients. Actinobacteria, Bacteroidetes, Firmicutes, and mOTUs were related to each metabotypes and cytokines by partial (age, sex, BMI and diet-corrected) Mantel tests. Edge width corresponded to the Mantel’s r statistic for the corresponding metabotypes and cytokines correlations, and edge color denoted the statistical significance based on 9,999 permutations. **(B)** Venn plot of the number of differentially abundant mOTUs that were associated with serum metabolites and cytokines, respectively. **(C)** *Clostridium symbiosum* contributed to C–C motif chemokine ligand 2 (CCL2) through cholic acid (P_mediation_ = 0.022, 20%). **(D)** *Eggerthella lenta* contributed to CCL2 through asparaginyl-valine (P_mediation_ = 0.012, 24%). **(E)** *Eggerthella lenta* contributed to CCL2 through cholic acid (P_mediation_ = 0.010, 24%). The gray lines indicated the Spearman correlations adjusting for age, sex, BMI and diet, with corresponding rho coefficients and false discovery rate values. The blue arrowed lines indicated the microbial effects on CCL2 mediated by metabolites, with the corresponding mediation *P-*values.

Concordantly, the Mantel test was performed on DA bacterial phylum between the cases and controls to identify phylum-related metabotypes. Notably, all three phyla and total mOTUs were significantly correlated with M 1, 10, and 14, which were mainly fatty acyls, glycerophospholipids, and dicarboxylic acids (**Figure 4A**). The correlation trends of Bacteroidetes and total mOTU with metabolites were more similar, suggesting that Bacteroidetes may play a more important role in regulating metabolism than other bacteria. In addition, we found that gut bacteria showed significantly more correlations with serum metabolites than with serum cytokines. Except for the significant positive correlation between CAG11 and CCL2 (**Figure 3B**), we did not find any CAGs directly correlated with these six cytokines. In contrast, serum metabolites were significantly correlated with the levels of inflammatory markers. Moreover, SCZ-enriched metabotypes were positively correlated with the pro-inflammatory cytokines, whereas HC-enriched metabotypes were inversely correlated (**Figure 3A** and **Table S6**).

Of the 31 mOTUs associated with metabolites or cytokines, two mOTUs in CAG11 correlated with both metabolites and cytokines (**Figure 4B**). To further evaluate the role of metabolites in the regulation of microbial effects on cytokines, we performed directional mediation analysis and found three mediation linkages. For instance, *Clostridium symbiosum* may contribute to increased CCL2 levels by decreasing serum cholic acid levels (**Figure 4C**), and *Eggerthella lenta* may contribute to increased CCL2 levels by affecting serum levels of cholic acid and asparaginyl-valine (**Figures 4D, E** **)**. Subsequently, we conducted additional validation based on 23 SCZ patients and 23 controls and found that cholic acid may mediate the association between bacteria and CCL2, but the result did not reach statistical difference due to the limited sample size (**Figures S7B, C**). The interaction networks between CAGs, SCZ-associated metabolites, and cytokines suggest that dysbiotic gut bacteria may alter inflammatory cytokines by interacting with host metabolites.

### SCZ Diagnosis Based on the Metabolites Related to the Gut Microbiota

**Figure 5:**
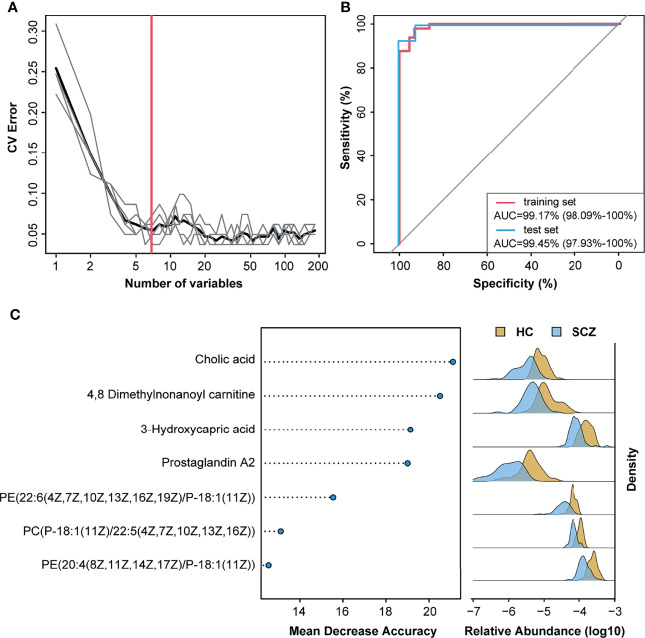
Metabolites classify schizophrenia from healthy control. **(A)** Distribution of five trials of 10-fold cross-validation error in random forest classification of schizophrenia as the number of features increases. The model was constructed using relative abundance of the 50 metagenomic operational taxonomic units and 133 metabolites from 70% of schizophrenia and healthy control samples (training set). The black curve indicates the average of the five trials (gray lines). The pink line marks the number of features in the optimal set. **(B)** Receiver operating characteristic analysis was performed to evaluate the diagnostic performance of these seven metabolite biomarkers, and the areas under the curve of the training set and test set were 99.17% and 99.45%, respectively. **(C)** The seven metabolites with most weight to discriminate schizophrenia and controls were selected by the cross-validated random forest models. The length of line indicated the importance of the metabolites for classification.

Previous studies have established a diagnosis model of SCZ using only single omics data, the gut microbiota, or metabolites. To improve the diagnostic efficiency, we combined 50 DA mOTUs and 133 serum metabolites to construct disease classifiers. In the random forest cross-validation within the training set, the classification error of seven serum metabolites was the lowest, and the areas under the ROC curve of the training set and test set were 99.17% and 99.45%, respectively. (**Figures 5A, B** **)**. Among the discriminatory features included in the classifier, cholic acid had the greatest impact, followed by metabolites such as 4,8-dimethylnonanoyl carnitine, 3-hydroxycapric acid, and prostaglandin A2 (**Figure 5C**). All metabolites in the model were significantly reduced in the SCZ group. Moreover, with the exception of 4,8-dimethylnonanoyl carnitine, the other six metabolites showed a significant negative correlation with the Shannon index (**Table S7**). Reduced cholic acid levels in the serum of SCZ patients were also verified in another study (**Figure S7A**). Overall, serum metabolites showed better potential for diagnosing SCZ than gut bacteria, especially gut microbe-derived metabolites.

## Discussion

**Figure 6:**
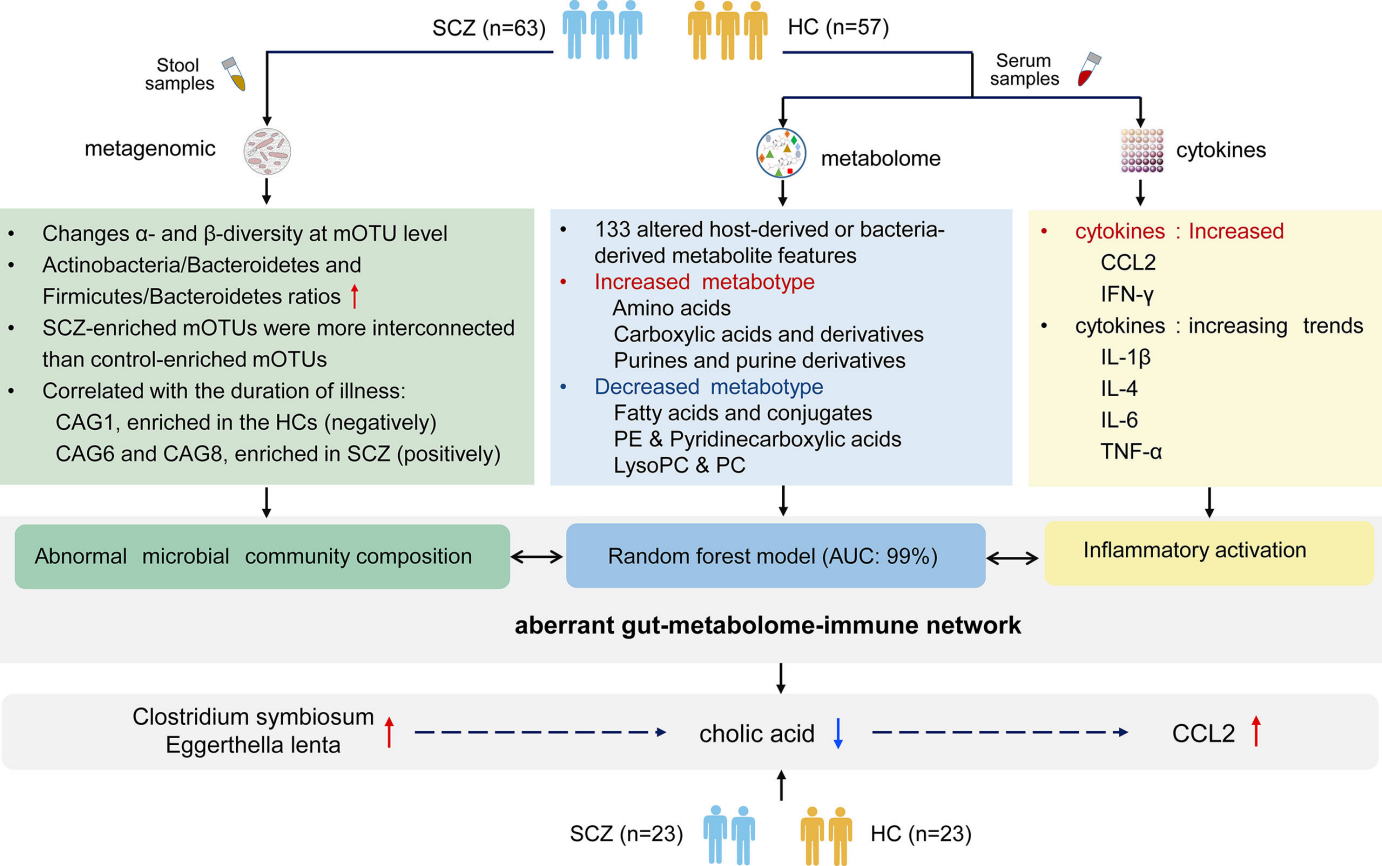
The summary of gut microbiota composition, metabolism and cytokines analysis between schizophrenia and healthy control.

As shown in summary of **Figure 6**, we demonstrated significant differences in serum metabolite profiles, intestinal microbiota, and aberrant blood cytokine levels in SCZ patients compared with HCs. We revealed that metabotypes were significantly correlated with gut microbial CAGs and inflammatory cytokines, and intestinal microbiota may influence immune responses by regulating host metabolic processes.

We identified 133 SCZ-associated metabolites through untargeted metabolome studies, some of which have been reported previously (33–38). Recent meta-analyses have highlighted the association between SCZ and increased amino acids, fatty acids, glycerophospholipids, and reduced PC/PE (14, 15, 39, 40), and most of the SCZ-relevant serum metabolites identified here were also in these categories. Furthermore, our data indicated that metabolites with similar chemical structures tended to cluster together, and those in the same cluster strongly correlated with each other. For example, a cluster of metabolites (M3) comprising seven types of amino acids, upregulated in the serum of the SCZ group, and M13, which included six metabolites belonging to glycerophosphocholines, was significantly reduced in the SCZ group. The formation of co-varying clusters of metabolites may be due to a variety of biochemical mechanisms, including chemical modifications of common parent metabolites, being associated with the same biochemical pathway, and being co-produced by specific microorganisms. Correlation analysis with the course of disease showed that some serum metabolites decreased with the progression of disease, such as MNA, a metabolite of M14. MNA can inhibit glutamate excitotoxicity and increase the level of acetylcholine in brain tissue (41, 42), providing a potential neuroprotective effect in neurodegenerative diseases and possibly a therapeutic target in the future.

Although the inflammatory hypothesis of SCZ has long been proposed (1, 2), the cause of long-term immune activation in SCZ remains unclear. Both gut microbes and serum metabolites are crucial regulators of host immune homeostasis (43, 44). The correlations between inflammatory cytokines and serum metabolites appear to be stronger and more widely presented than those with gut microbes, suggesting that serum metabolites may play a more direct role in the inflammatory process of SCZ than gut microbiota. Accordingly, some metabolites associated with bioenergetics or biosynthesis have emerged as immune effector molecules, with specific roles in the control of the immune system (45). In the present study, PEs, PCs, and sphingolipids were negatively correlated with inflammatory cytokines, whereas amino acid metabolites were positively correlated with these cytokines. Moreover, SCZ patients expressed higher levels of metabolites that carry pro-inflammatory potential, such as asymmetric dimethylarginine (46), and had depleted anti-inflammatory metabolites, such as oleic acid and arachidonic acid (19, 20). The imbalance of immune-regulatory metabolites in the serum of SCZ patients may contribute to their systemic immune activation.

The human intestinal microbiota interacts extensively with the host through substrate co-metabolism and metabolic exchange (47, 48). Our results showed that SCZ-relevant bacteria were significantly associated with amino acids, fatty acids, and glycerophospholipids. Interestingly, these metabolites are partially involved in the metabolism of neurotransmitters and the regulation of neuroinflammation (18, 49, 50). Consistent with previous studies, we found significant increases in the relative abundance of Bacteroides, Collinsella, Clostridium, and Dialister and a decrease in Klebsiella and Streptococcus at the genus level (8, 51). It should be noted that significantly enriched *Akkermansia muciniphila* and *Streptococcus vestibularis* in SCZ were reported in our previous study (9); however, this analysis did not show significant differences between groups. These inconsistencies may be influenced by the highly heterogeneous characteristics of the disease, sample size, and other potential confounding factors. But it was certain that most bacteria, including these two, changed in the same direction in SCZ across our two cohorts. In addition, we also found that the microbial composition of SCZ patients displayed a higher α-diversity score. Although a more diverse microbiota is generally considered to be healthier (52), higher α-diversity above a threshold is associated with unhealthy blood levels of specific microbial metabolites (13). For example, α-diversity is elevated in people with constipation (53), and we found more pronounced constipation in SCZ patients, consistent with previous reports (54). Notably, the metabolites associated with lipid and energy metabolism were associated with Shannon index, demonstrating a strong connection between host physiology and gut microbiota structure in SCZ patients. By constructing random forest model based on omics data, we identified that metabolites, especially those significantly associated with α-diversity and cytokines, were more suitable as biomarkers than microbiota. Compared with previous microbial diagnostic models (8, 9), the diagnostic accuracy of seven metabolites can reach 99%. And these metabolites (such as cholic acid and prostaglandin A2) can be studied in the future to better understand their roles in the pathogenesis and pathophysiology of SCZ.

Ecologically, gut bacteria work as functional groups, flourishing or declining together in response to the changes of physiological environment, rather than in isolation (30, 55). Therefore, CAG-based analysis in our study provides a more ecologically relevant approach to reduce the dimension of the microbiota dataset and facilitates to identify functionally important members of the intestinal microbiota in SCZ. Similarly, we also found that bacteria with higher homology were more easily clustered together, indicating that their symbiosis ability was stronger. These CAG clusters also provide some clues for a more detailed taxonomic classification of unannotated mOTUs. The abundance of CAG11 increased in SCZ and was positively correlated with the chemokine CCL2. Clostridium, an important genus in this CAG, has also previously been reported to upregulate CCL2 and various pro-inflammatory cytokines in mice (56). Moreover, Clostridium was also found to be positively correlated with amino acids in inflammatory bowel disease (57), consistent with our results. Notably, we did not find any other significant correlations between CAGs and these cytokines, suggesting that bacteria may indirectly modulate immune activation in SCZ patients. We also confirmed this hypothesis through intermediate analysis; for example, microbes may affect inflammatory cytokines by regulating metabolic processes, such as bile acid metabolism. Previous studies have shown intestinal communication between bile acids and microbiota, also its effects on host immune responses (58, 59). Considering the dynamic nexus of host immunological, metabolic, and microbial networks, the case-control study is insufficient to depict the overall disease status; long-term follow-up and functional studies are urgently needed. It is more likely to reveal specific bacteria that may be responsible for SCZ immune abnormalities through the production of bioactive metabolites. In addition, the inconsistency of several gut microbiota studies on SCZ also reflects the limitation of research method. Thus, it is necessary to expand the sample size and optimize analysis method to accurately describe gut microbiota features for SCZ in detail.

Overall, through multi-omics correlation analysis, we found several metabotypes closely related to gut microbial metabolism and showed significant correlations with serum inflammatory cytokines in SCZ patients. More studies using multi-omics analysis to gain a systematic understanding of the mechanisms of body synergistic regulation will deepen insight into the systems biology of SCZ and promote the development of personalized multimode SCZ intervention. In addition, it has been confirmed that disorders in bile acid metabolism may contribute to cognitive changes (60), and our study also demonstrated the importance of cholic acid in regulating the relationship between microbes and immunity. The role of bile acid metabolism in the gut brain axis needs be further investigated in the future, and the gut microbiota-bile acid metabolism pathway-immune activation could be possible therapeutic targets for schizophrenia.

## Data Availability Statement

The datasets presented in this study can be found in online repositories. The names of the repository/repositories and accession number(s) can be found below: https://bigd.big.ac.cn/gsa/browse/CRA004662, CRA004662.

## Ethics Statement

The studies involving human participants were reviewed and approved by The Ethics Committee of the First Affiliated Hospital of Xi’an Jiaotong University. The patients/participants provided their written informed consent to participate in this study.

## Author Contributions

XM and FZ conceived and designed project. QM, ZY, BZ, and XH collected samples. YF, YG, FG, and WW did experiments and analysis. YF, JY, BY, and LQ prepared Figures. All of authors performed data analyses and interpretations. YF and YG had full access to all the data in the study and take responsibility for the integrity of the data and the accuracy of the data analysis. All authors contributed to the article and approved the submitted version.

## Funding

This study was supported by the Key Research and Development Program of Shaanxi (No. 2020ZDLSF02-10), the National Natural Science Foundation of China (No. 81771471), Basic Research Project of Natural Science Fund of Shaanxi Province (No. 2016JQ8026), the Outstanding Youth Science Foundation Project of National Natural Science Foundation of China (No. 82022023).

## Conflict of Interest

The authors declare that the research was conducted in the absence of any commercial or financial relationships that could be construed as a potential conflict of interest.

## Publisher’s Note

All claims expressed in this article are solely those of the authors and do not necessarily represent those of their affiliated organizations, or those of the publisher, the editors and the reviewers. Any product that may be evaluated in this article, or claim that may be made by its manufacturer, is not guaranteed or endorsed by the publisher.
