# Supplementary figures and images for: Multi-Omics Analysis Reveals Aberrant Gut-Metabolome-Immune Network in Schizophrenia
Source: Front Immunol. 2022 Mar 3;13:812293. doi: 10.3389/fimmu.2022.812293 (PMC8927969; doi:10.3389/fimmu.2022.812293)

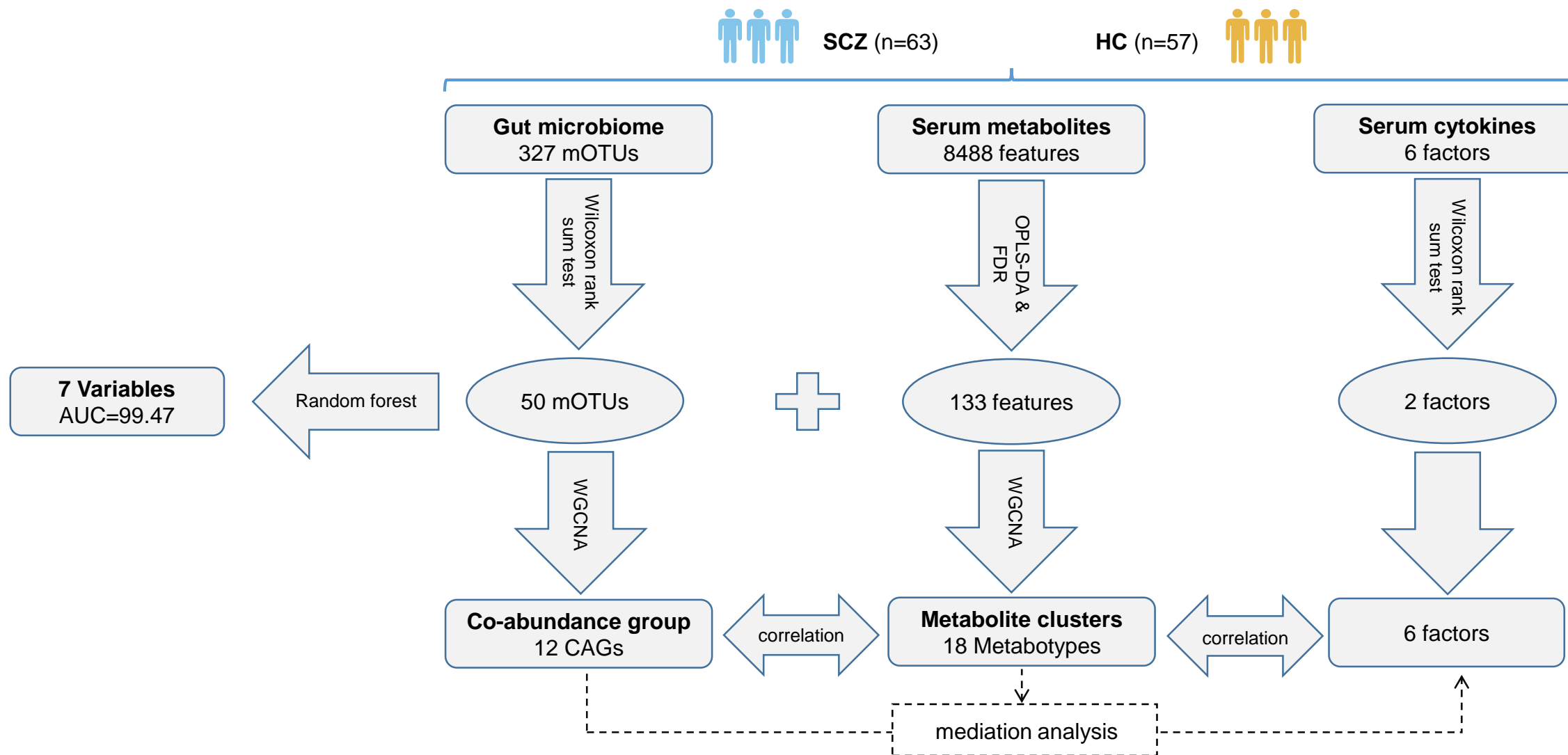

Supplement: Supplementary file 1 [file Image_1.pdf]

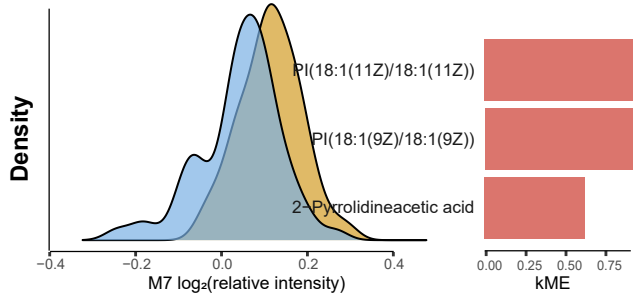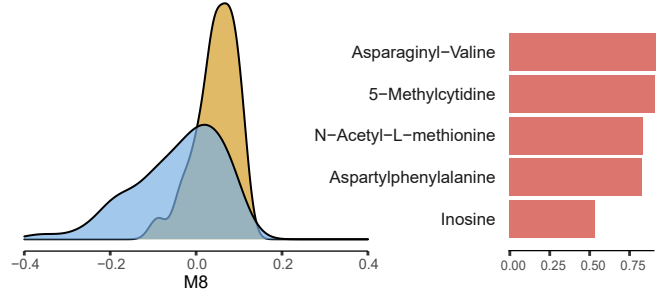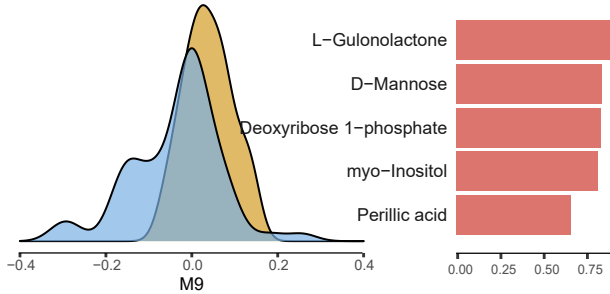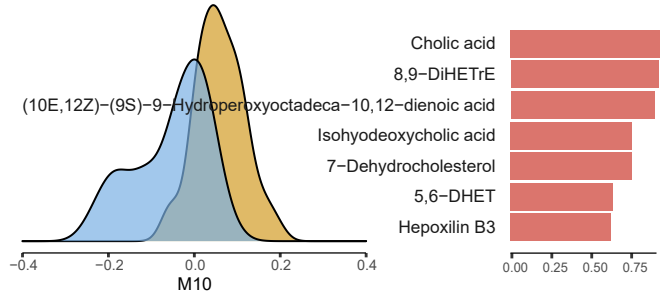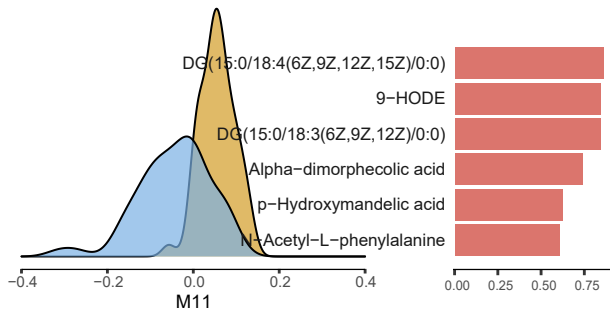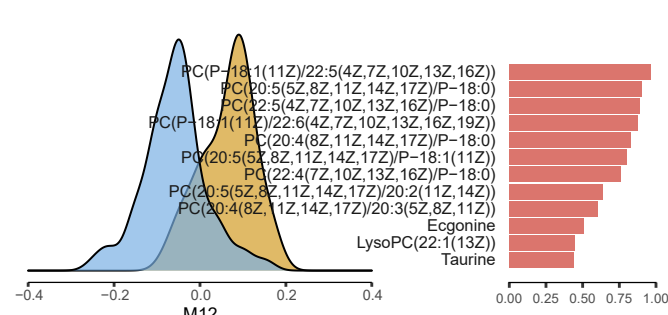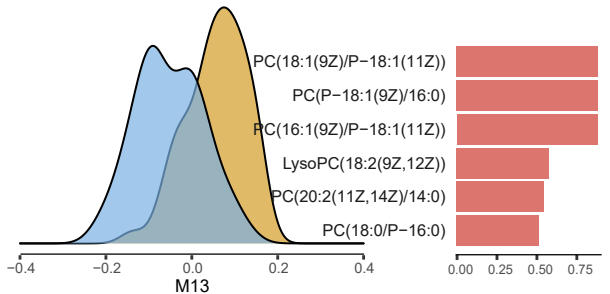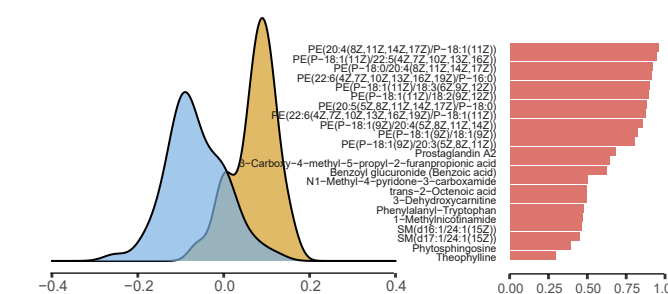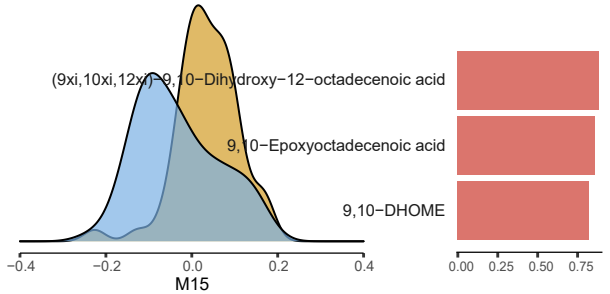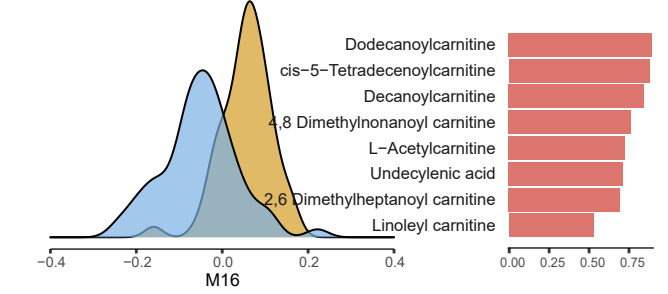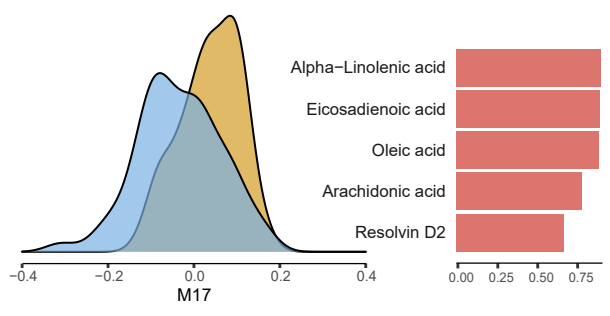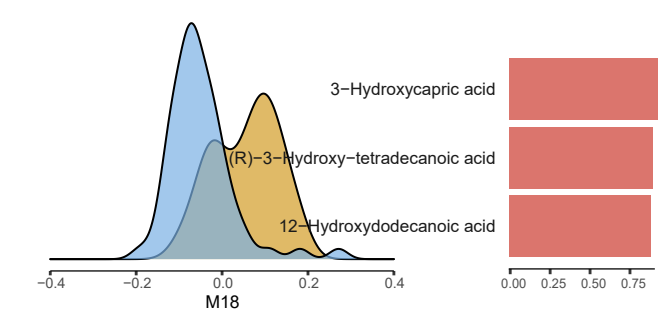

Supplement: Supplementary file 3 [file Image_3.pdf]

Density

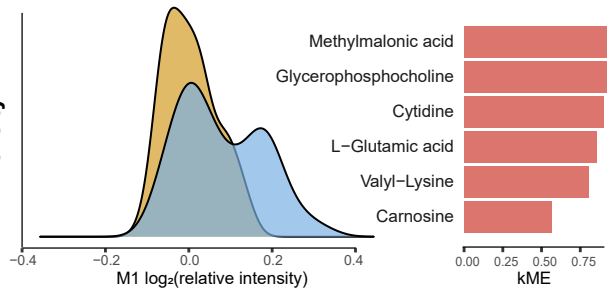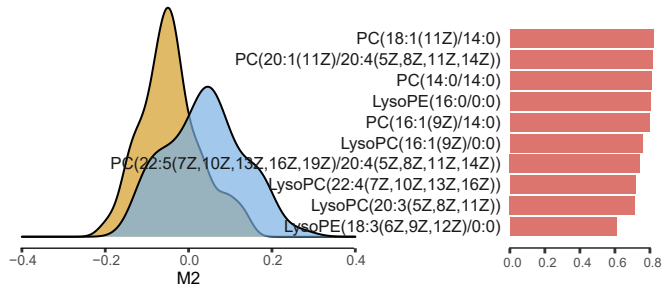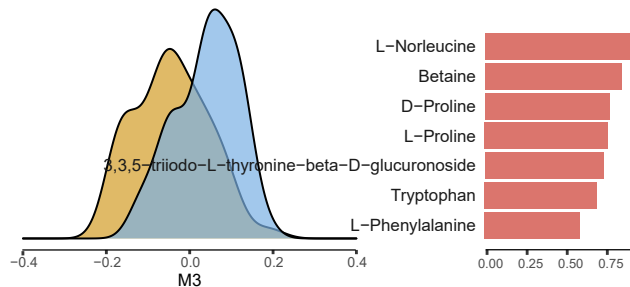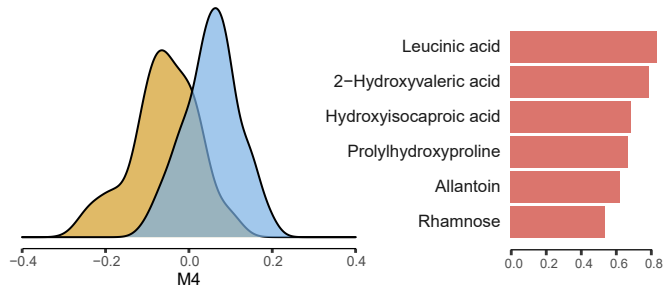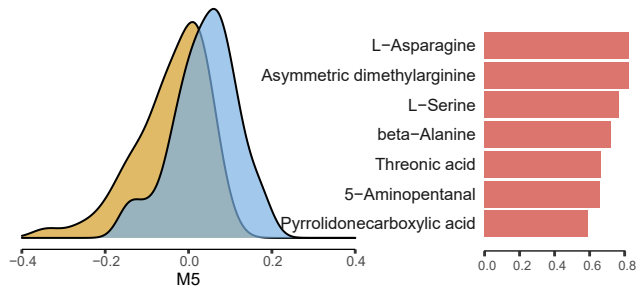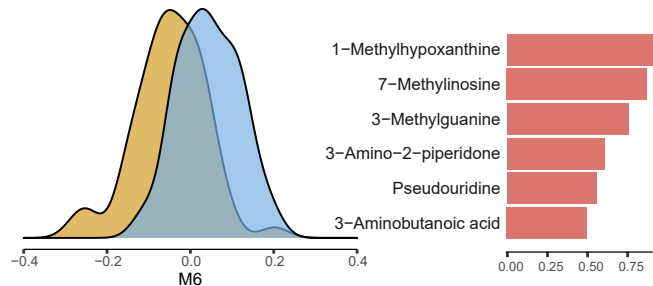

Supplement: Supplementary file 4 [file Image_4.pdf]

**A**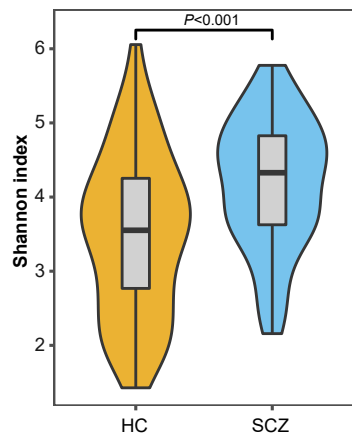**B**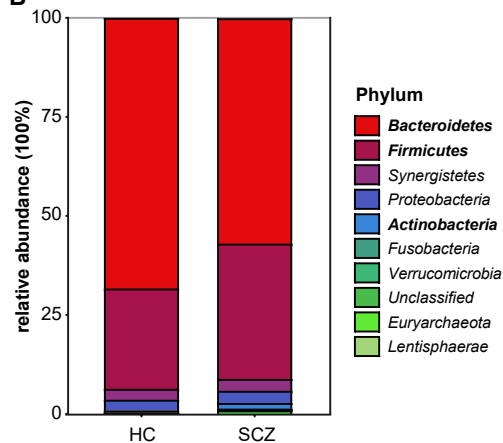**C**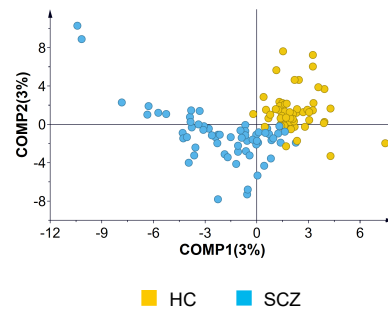**D**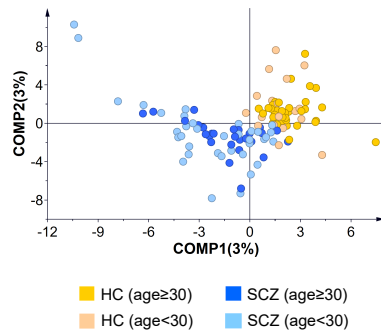**E**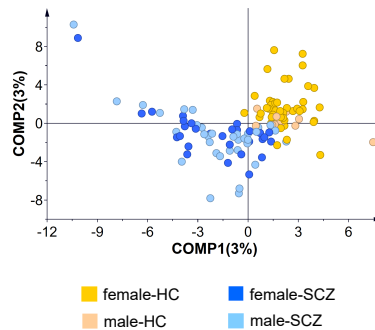**F**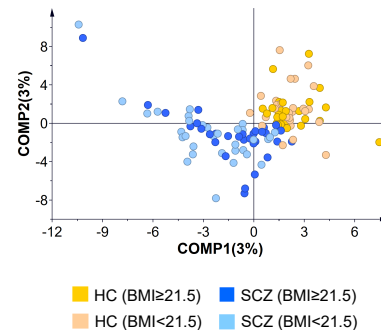**G**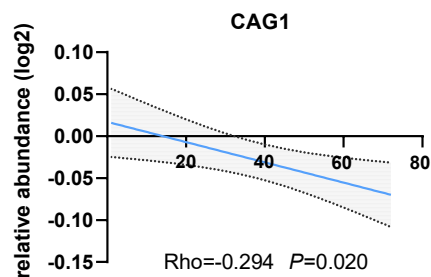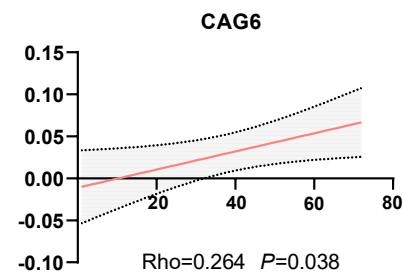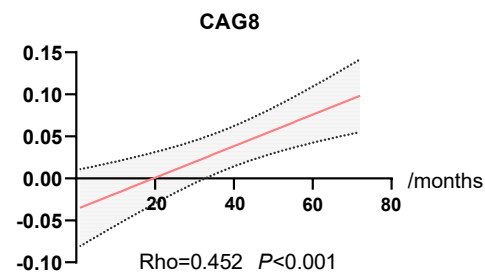

Duration of illness

Supplement: Supplementary file 5 [file Image_5.pdf]

**A**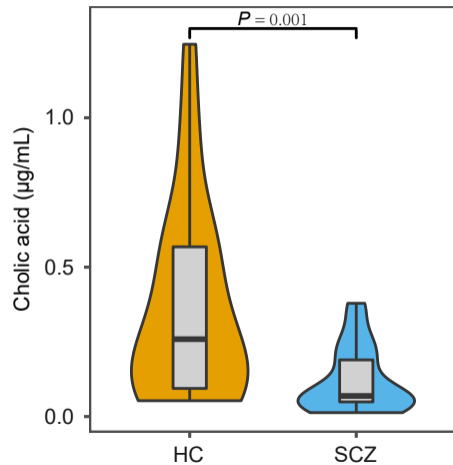**B**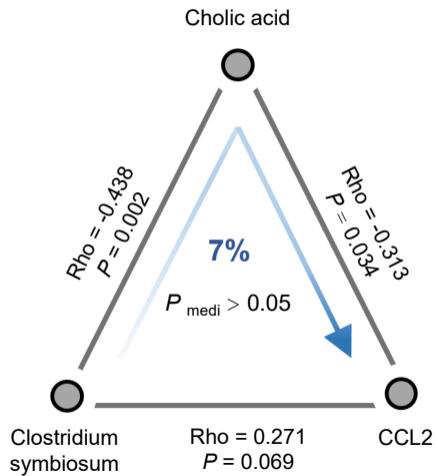**C**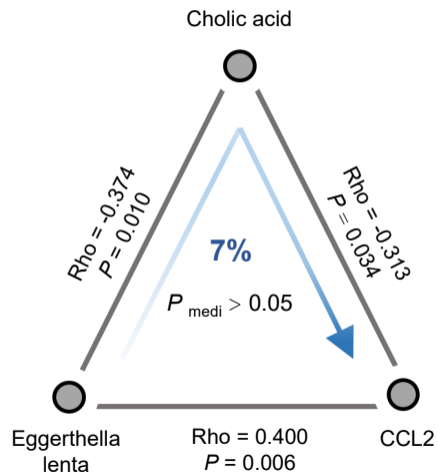

Supplement: Supplementary file 7 [file Image_7.pdf]
