# Supplementary material for: Multi-Omics Analysis Reveals Aberrant Gut-Metabolome-Immune Network in Schizophrenia
Source: Front Immunol. 2022 Mar 3;13:812293. doi: 10.3389/fimmu.2022.812293 (PMC8927969; doi:10.3389/fimmu.2022.812293)
Supplement: Supplementary file 2 [file Image_2.pdf]

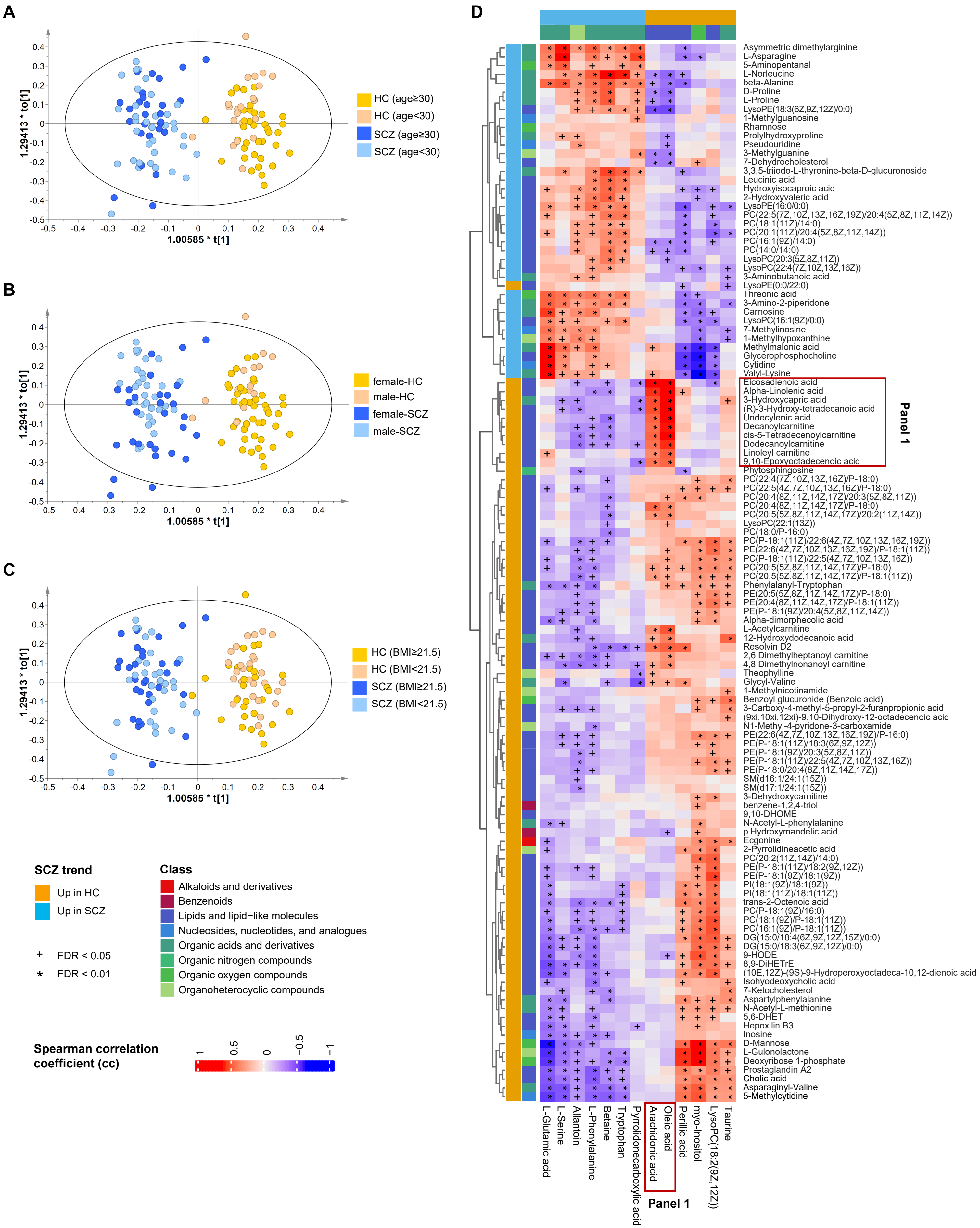
A
B
C
D
1.29413 * to[1]
1.00585 * t[1]
HC (age≥30)
HC (age<30)
SCZ (age≥30)
SCZ (age<30)
female-HC
male-HC
female-SCZ
male-SCZ
HC (BMI≥21.5)
HC (BMI<21.5)
SCZ (BMI≥21.5)
SCZ (BMI<21.5)
SCZ trend
Up in HC
Up in SCZ
Class
Alkaloids and derivatives
Benzenoids
Lipids and lipid−like molecules
Nucleosides, nucleotides, and analogues
Organic acids and derivatives
Organic nitrogen compounds
Organic oxygen compounds
Organoheterocyclic compounds
+ FDR < 0.05
* FDR < 0.01
Spearman correlation coefficient (cc)
1
0.5
0
−0.5
−1
Asymmetric dimethylarginine
L-Asparagine
5-Aminopentanal
L-Norleucine
beta-Alanine
D-Proline
L-Proline
LysoPE(18:3(6Z,9Z,12Z)/0:0)
1-Methylguanosine
Rhamnose
Prolylhydroxyproline
Pseudouridine
3-Methylguanine
7-Dehydrocholesterol
3,3,5-triiodo-L-thyronine-beta-D-glucuronoside
Leucinic acid
Hydroxyisocaproic acid
2-Hydroxyvaleric acid
LysoPE(16:0/0:0)
PC(22:5(7Z,10Z,13Z,16Z,19Z)/20:4(5Z,8Z,11Z,14Z))
PC(18:1(11Z)/14:0)
PC(20:1(11Z)/20:4(5Z,8Z,11Z,14Z))
PC(16:1(9Z)/14:0)
PC(14:0/14:0)
LysoPC(20:3(5Z,8Z,11Z))
LysoPC(22:4(7Z,10Z,13Z,16Z))
3-Aminobutanoic acid
LysoPE(0:0/22:0)
Threonic acid
3-Amino-2-piperidone
Carnosine
LysoPC(16:1(9Z)/0:0)
7-Methylinosine
1-Methylhypoxanthine
Methylmalonic acid
Glycerophosphocholine
Cytidine
Valyl-Lysine
Eicosadienoic acid
Alpha-Linolenic acid
3-Hydroxycapric acid
(R)-3-Hydroxy-tetradecanoic acid
Undecylenic acid
Decanoylcarnitine
cis-5-Tetradecenoylcarnitine
Dodecanoylcarnitine
Linoleyl carnitine
9,10-Epoxyoctadecenoic acid
Panel 1
Phytosphingosine
PC(22:4(7Z,10Z,13Z,16Z)/P-18:0)
PC(22:5(4Z,7Z,10Z,13Z,16Z)/P-18:0)
PC(20:4(8Z,11Z,14Z,17Z)/20:3(5Z,8Z,11Z))
PC(20:4(8Z,11Z,14Z,17Z)/P-18:0)
PC(20:5(5Z,8Z,11Z,14Z,17Z)/20:2(11Z,14Z))
LysoPC(22:1(13Z))
PC(18:0/P-16:0)
PC(P-18:1(11Z)/22:6(4Z,7Z,10Z,13Z,16Z,19Z))
PE(22:6(4Z,7Z,10Z,13Z,16Z,19Z)/P-18:1(11Z))
PC(P-18:1(11Z)/22:5(4Z,7Z,10Z,13Z,16Z))
PC(20:5(5Z,8Z,11Z,14Z,17Z)/P-18:0)
PC(20:5(5Z,8Z,11Z,14Z,17Z)/P-18:1(11Z))
Phenylalanyl-Tryptophan
PE(20:5(5Z,8Z,11Z,14Z,17Z)/P-18:0)
PE(20:4(8Z,11Z,14Z,17Z)/P-18:1(11Z))
PE(P-18:1(9Z)/20:4(5Z,8Z,11Z,14Z))
Alpha-dimorphecolic acid
L-Acetylcarnitine
12-Hydroxydodecanoic acid
Resolvin D2
2,6 Dimethylheptanoyl carnitine
4,8 Dimethylnonanoyl carnitine
Theophylline
Glycyl-Valine
1-Methylnicotinamide
Benzoyl glucuronide (Benzoic acid)
3-Carboxy-4-methyl-5-propyl-2-furanpropionic acid
(9xi,10xi,12xi)-9,10-Dihydroxy-12-octadecenoic acid
N1-Methyl-4-pyridone-3-carboxamide
PE(22:6(4Z,7Z,10Z,13Z,16Z,19Z)/P-16:0)
PE(P-18:1(11Z)/18:3(6Z,9Z,12Z))
PE(P-18:1(9Z)/20:3(5Z,8Z,11Z))
PE(P-18:1(11Z)/22:5(4Z,7Z,10Z,13Z,16Z))
PE(P-18:0/20:4(8Z,11Z,14Z,17Z))
SM(d16:1/24:1(15Z))
SM(d17:1/24:1(15Z))
3-Dehydroxycarnitine
benzene-1,2,4-triol
9,10-DHOME
N-Acetyl-L-phenylalanine
p.Hydroxymandelic.acid
Ecgonine
2-Pyrrolidineacetic acid
PC(20:2(11Z,14Z)/14:0)
PE(P-18:1(11Z)/18:2(9Z,12Z))
PE(P-18:1(9Z)/18:1(9Z))
PI(18:1(9Z)/18:1(9Z))
PI(18:1(11Z)/18:1(11Z))
trans-2-Octenoic acid
PC(P-18:1(9Z)/16:0)
PC(18:1(9Z)/P-18:1(11Z))
PC(16:1(9Z)/P-18:1(11Z))
DG(15:0/18:4(6Z,9Z,12Z,15Z)/0:0)
DG(15:0/18:3(6Z,9Z,12Z)/0:0)
9-HODE
8,9-DiHETrE
(10E,12Z)-(9S)-9-Hydroperoxyoctadeca-10,12-dienoic acid
Isohyodeoxycholic acid
7-Ketocholesterol
Aspartylphenylalanine
N-Acetyl-L-methionine
5,6-DHET
Hepoxilin B3
Inosine
D-Mannose
L-Gulonolactone
Deoxyribose 1-phosphate
Prostaglandin A2
Cholic acid
Asparaginyl-Valine
5-Methylcytidine
L-Glutamic acid
L-Serine
Allantoin
L-Phenylalanine
Betaine
Tryptophan
Pyrrolidonecarboxylic acid
Arachidonic acid
Oleic acid
Perillic acid
myo-Inositol
LysoPC(18:2(9Z,12Z))
Taurine
Panel 1
