# Supplementary material for: Multi-Omics Analysis Reveals Aberrant Gut-Metabolome-Immune Network in Schizophrenia
Source: Front Immunol. 2022 Mar 3;13:812293. doi: 10.3389/fimmu.2022.812293 (PMC8927969; doi:10.3389/fimmu.2022.812293)
Supplement: Supplementary file 6 [file Image_6.pdf]

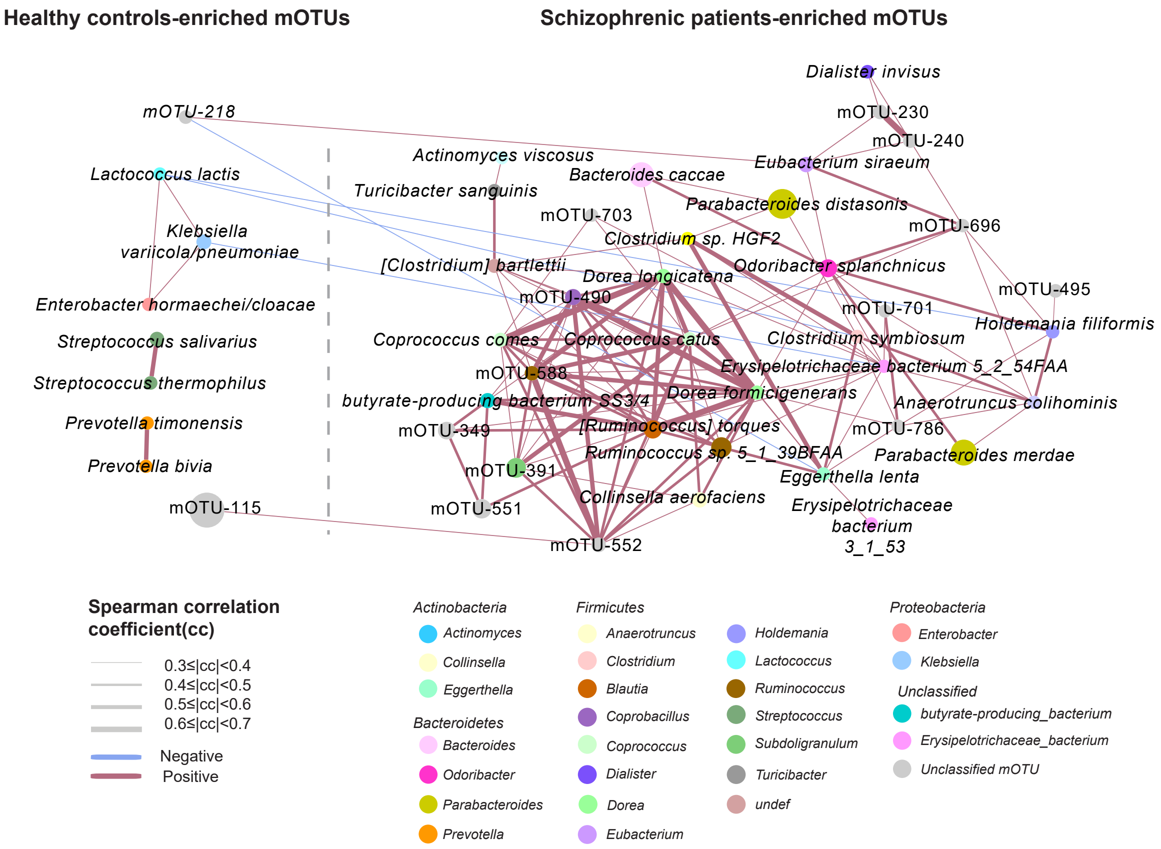

Healthy controls-enriched mOTUs
Schizophrenic patients-enriched mOTUs
mOTU-218
Lactococcus lactis
Klebsiella variicola/pneumoniae
Enterobacter hormaechei/cloacae
Streptococcus salivarius
Streptococcus thermophilus
Prevotella timonensis
Prevotella bivia
mOTU-115
Dialister invisus
mOTU-230
mOTU-240
Eubacterium siraeum
Actinomyces viscosus
Bacteroides caccae
Turicibacter sanguinis
Parabacteroides distasonis
mOTU-703
mOTU-696
Clostridium sp. HGF2
Odoribacter splanchnicus
[Clostridium] bartlettii
Dorea longicatena
mOTU-490
mOTU-495
mOTU-701
Holdemania filiformis
Coprococcus comes
Coprococcus catus
Clostridium symbiosum
mOTU-588
Erysipelotrichaceae bacterium 5_2_54FAA
butyrate-producing bacterium SS3/4
Dorea formicigenerans
Anaerotruncus colihominis
mOTU-349
[Ruminococcus] torques
mOTU-786
Ruminococcus sp. 5_1_39BFAA
Parabacteroides merdae
mOTU-391
Eggerthella lenta
Collinsella aerofaciens
mOTU-551
Erysipelotrichaceae bacterium 3_1_53
mOTU-552
Spearman correlation coefficient(cc)
0.3≤|cc|<0.4
0.4≤|cc|<0.5
0.5≤|cc|<0.6
0.6≤|cc|<0.7
Negative
Positive
Actinobacteria
Actinomyces
Collinsella
Eggerthella
Bacteroidetes
Bacteroides
Odoribacter
Parabacteroides
Prevotella
Firmicutes
Anaerotruncus
Clostridium
Blautia
Coprobacillus
Coprococcus
Dialister
Dorea
Eubacterium
Holdemania
Lactococcus
Ruminococcus
Streptococcus
Subdoligranulum
Turicibacter
undef
Proteobacteria
Enterobacter
Klebsiella
Unclassified
butyrate-producing_bacterium
Erysipelotrichaceae_bacterium
Unclassified mOTU
