# Supplementary material for: Multi-Omics Analysis Reveals Aberrant Gut-Metabolome-Immune Network in Schizophrenia
Source: Front Immunol. 2022 Mar 3;13:812293. doi: 10.3389/fimmu.2022.812293 (PMC8927969; doi:10.3389/fimmu.2022.812293)
Supplement: Supplementary file 9 [file Presentation_1.pdf]

Supplementary Material

Supplementary Figure 1. Flowchart of the study analyses in this study.

Supplementary Figure 2. Metabolomic analysis of serum samples from schizophrenic patients and healthy controls. (**A-C)** The global metabolites were not clustered based on age, sex, or BMI. The age and BMI were grouped according to the medians of total subjects, respectively. (**D)** Spearman correlations between newly discovered metabolites and 13 previously reported metabolites. The color represented positive (red) or negative (blue) correlations, and false discovery rates (FDRs) are denoted as follows: +FDR < 0.05, *FDR < 0.01.

Supplementary Figure 3. Metabotypes significantly decreased in the schizophrenia group. Relative intensity distributions of significantly decreased metabotypes (clusters) in the SCZ group compared to the healthy control group. The length of the bar in the histogram represented the kME value, which indicated the biweight midcorrelation between the metabolite profile and module eigenvector.

Supplementary Figure 4. Metabotypes significantly increased in the schizophrenia group. Relative intensity distributions of six metabotypes (clusters) significantly elevated in the SCZ group. The detailed metabolites based on the weighted gene co-expression network analysis were presented on the right side of each density plot. The length of the bar in the histogram represented the kME value, which indicated the biweight midcorrelation between the metabolite profile and module eigenvector.

Supplementary Figure 5. Increased gut microbiome diversity in schizophrenia. **(A)** α-diversity (Shannon index) between the two groups at the metagenomic operational taxonomic unit (mOTU) level. *P*-values from the Wilcoxon rank-sum test were shown. (**B)** The relative abundance of the two groups at the phylum level. The names of bacteria with significant differences between the two groups were bolded. (**C)** At the mOTU level, partial least squares discriminant analysis showed that gut microbiota composition of SCZ patients was greatly different from that of healthy controls. (**D-F)** The global microbial phenotypes (at the mOTU level) were not clustered based on age, sex, and body mass index (BMI). The age and BMI were grouped according to the medians of total subjects, respectively. (**G)** Spearman correlations between co-abundance groups and duration of illness.

Supplementary Figure 6. Network of metagenomic operational taxonomic units differentially enriched in schizophrenic patients vs. healthy controls. For all mOTUs present in more than 5% of the samples, the direction of enrichment was determined by the Wilcoxon rank-sum test (*P*-value < 0.05). Node sizes reflected the mean abundance of significant mOTUs. mOTUs annotated to species were colored according to genus (red edges, Spearman correlation coefficient > 0.3, *P*-value < 0.05; blue edges,

Spearman correlation coefficient < −0.3, *P*-value < 0.05).

Supplementary Figure 7. Cholic acid was further validated by targeted liquid chromatography-mass spectrometry. **(A)** Violin plot of level of cholic acid between the schizophrenia and healthy control groups. **(B–C)** Correlation between *Clostridium symbiosum*/*Eggerthella lenta*, cholic acid, and CCL2.

Supplementary Table 1. Clinical characteristic of the schizophrenic patients and healthy controls.

Supplementary Table 2. 133 differentially abundant (DA) metabolites between the two groups.

Supplementary Table 3. The composition of the 18 fasting serum metabolite clusters making up the SCZ-enriched and HC-enriched metabotypes in 120 subjects.

Supplementary Table 4. Differences of bacteria at phylum level between the two groups.

Supplementary Table 5. Significantly differentially enriched mOTUs between SCZ patients and healthy controls.

Supplementary Table6. Multi-omics analysis of gut microbiome, metabolomics and cytokines.

Supplementary Table 7. Key serum metabolites responsible for distinguishing SCZ patients from HCs.

Code availability

#Correlation analysis

```
library(ppcor)
library(MASS)
##Read the data
t2<-read.csv("test.csv", header = T)
t2[is.na(t2)]<- 0
##Create a new vector
rnormtest2 <- data.frame()
##x:t2, ab:clinical data, cd:flora, ef:correction
pcor.spearman<-function(x,a,b,c,d,e,f){for (i in a:b){
  for (j in c:d){

    re = pcor.test(x[,i],x[,j],x[,c(e:f)],method="spearman");
    rnormtest2 <- rbind(rnormtest2, re)
  }
};
  for (i in a:b){
    for (j in c:d){
      lab = paste(names(x)[i],names(x)[j]);

      rnormtest2 <- rbind(rnormtest2, lab)
    }
  };
  ac<-nrow(rnormtest2);
  ta<-ac/2;
  tb<-rnormtest2[ta+1:ac,];
  rnormtes<-rnormtest2[1:ta,];
  rnormtes<-cbind(rnormtes,tb[,1]);
  student<-rnormtes$`tb[, 1]`;
  student<-as.character(student);
  name<-strsplit((student)," ");
  WMH<-sapply(name,"[",1);
  genus<-sapply(name,"[",2);
  rnormtest<-cbind(WMH,genus,rnormtes[,-7]);
  rnormtest<-rnormtest[1:ta,];rnormtest}
result<-pcor.spearman(t2,21,29,14,20,3,6)
result.rho<- result[,1:3]
result.p<-result[,c(1,2,4)]
#data convert to numerical type
result.rho$estimate<-as.numeric(result.rho$estimate)
write.csv(result.rho,file="r.csv")
result.p$p.value<-as.numeric(result.p$p.value)
result.p.fdr<-p.adjust(result.p$p.value, method="fdr",n=length(result.p$p.value))
```

```
result.p.fdr1<-data.frame(result.p.fdr)
result.p.adj<-cbind(result.p,result.p.fdr1)
result.p.adj<-result.p.adj[,-3]
write.csv(result.p.adj,file="p.csv")
#data transform into a matrix
library(reshape2)
result.rhom<-dcast(result.rho,WMH~genus)
result.p<-dcast(result.p.adj,WMH~genus)
rownames(result.rhom)<-result.rhom[,1]
result.rhom<-result.rhom[,-1]
rownames(result.p)<-result.p[,1]
result.p<-result.p[,-1]
#draw
library(pheatmap)
data_mark=result.p
for(i in 1:7){for( j in 1:9){data_mark[i,j]=ifelse(result.p[i,j]<0.05,"*", " ")}}
pheatmap(result.rhom,
         display_numbers=data_mark,
         border_color="grey", legend_labels="rho")
```

#Density figure

```
library(ggplot2)
data2<-read.csv("data.csv", header = T, row.names = 1)
ggplot(data2, aes(x = M18))+
  geom_density(aes(fill = group),alpha=0.7)+
  theme_tufte()+
  scale_fill_manual(values = c("SCZ" = "#56B4E9", "HC" = "#E69F00"))+
  scale_x_continuous(limits = c(-0.4,0.4))
data5<-read.csv("7metabolite_for_density_plot.csv", header = T)
data6<-log10(data5[,2:8])
write.csv(data6,"data6.csv")
data7<-read.csv("data6.csv", header = T)
ggplot(data5, aes(x = Prostaglandin.A2))+
  geom_density(aes(fill = group),alpha=0.7)+
  theme_tufte()+
  scale_fill_manual(values = c("SCZ" = "#56B4E9", "HC" = "#E69F00"))
#scale_x_continuous(limits = c(-0.4,0.4))
```

#WGCNA

```
####input data：
#1. Metabolite data.csv, row name: sample name, column name: metabolite variable；
#2. mOTU data.csv, row name: sample name, column name: bacterial variable；
#3. phenotype data.csv， row name: sample name, column name: phenotype variable；
```

####output data：
#1. Metabolite clustering data: new metabolite module data after dimensionality reductions；
#2. Microbial clustering data: new microbial module data after dimension reduction；
#3.The calculation results of metabolite raw data, including the metabolite module to which each metabolite belongs, the correlation with the module (KME), the connectivity or importance within the module (KIN), and the correlation with representability (R, P, FDR);
#4. The calculation results of the original microbial data, including the microbial module to which each microorganism belongs, the correlation with the module (KME), the connectivity or importance within the module (KIN), and the correlation with representability (R, P, FDR);
#5. Correlation between metabolite modules and phenotypes；
#6. Correlation between microbial modules and phenotypes；
#7. Correlation between microbial module and metabolite module.

```
### Step 1 - Set working directory
setwd ("./top")
# step2_load.libraries.R
#library (xlsx) ### Saving to spreadsheet
#library (data.table) ### Fast read of large files into R
library (WGCNA) ### -  Clustering software. Previously reported work done using v1.34
library (flashClust) ### Clustering software
library (ppcor) ### Partial Spearman correlations, for confounder analysis. Previously reported work done using v1.0
library (gplots) ### Plotting
library (cowplot) ### Plotting; to arrange several plots on the same page
library (ggplot2) ### Plotting
library (plyr) ### Data transformations
### Step 3 - Import input files
options (stringsAsFactors = FALSE)
### - phenotypes
phenotypes = read.csv (file = "phenotype.csv", row.names = 1, header = T, sep = ",")
### - metabolites
metabolites = read.csv (file = "metabolite_data.csv", row.names = 1, header = T, sep = ",")
### - cluster_mapping_file_m
#cluster_mapping_file_m = read.csv (file = "./data/cluster_mapping_file_m.csv", header = T, sep = ",", row.names = 1)
#cluster_mapping_file_m$label = sapply (rownames (cluster_mapping_file_m), function (x) paste (cluster_mapping_file_m [x, "New_Name"], cluster_mapping_file_m [x, "Description"], sep = ": "))
### Step 5 - Identify clusters of metabolites
```

```
###Settings for WGCNA generally
cor_method= "spearman" ### for association with clinical parameters
corFun_tmp= "bicor"
cluster_method= "average"
corOptions_list= list (use = 'pairwise.complete.obs')
corOptions_str= "use = 'pairwise.complete.obs'"
BH_pval_asso_cutoff = 0.05
NetworkType= "signed" ### Signed-network (as the PC1 and median profile does not
make sense as a summary measure of a cluster with anticorrelated metabolites.)
###Specify data and parameters
dat_tmp = log2 (metabolites) ### work in logarithmic space

### Settings for WGCNA on metabolites measurements
### Once these are established the steps below can be run
RsquareCut_val= 0.89 ### usually ranges 0.80-0.95 but requires inspecting curves
mergingThresh= 0.20 ### Maximum dissimilarity of module eigengenes (i.e. 1-
correlation) for merging modules.
minModuleSize= 3 ### minimum number of metabolites constituting a cluster
SoftPower= 13 ### beta-value, main parameter to optimize
### Calculate weighted adjacency matrix
A = adjacency (dat_tmp, power = SoftPower, type = NetworkType, corFnc =
corFun_tmp, corOptions = corOptions_str)
colnames (A) = rownames (A) = colnames (dat_tmp)
### Define dissimilarity based on topological overlap
dissTOM = TOMdist (A, TOMType = NetworkType)
colnames (dissTOM) = rownames (dissTOM) = colnames (dat_tmp)
### Hierarchical clustering
metaTree = flashClust (as.dist (dissTOM), method = cluster_method)
### Define modules by cutting branches
moduleLabels1 = cutreeDynamic (dendro = metaTree, distM = dissTOM, method =
"hybrid", deepSplit = 4, pamRespectsDendro = T, minClusterSize = minModuleSize)
moduleLabels1 = labels2colors (moduleLabels1)
### Automatically merge highly correlated modules
merge = mergeCloseModules (dat_tmp, moduleLabels1, corFnc = corFun_tmp,
corOptions = corOptions_list, cutHeight = mergingThresh)
### Determine resulting merged module colors
moduleLabels2 = merge$colors
### Establish eigengenes of the newly merged modules, used for cluster overall
abundances
MEs = merge$newMEs
### Choose final module assignments
moduleColorsMeta = moduleLabels2
names (moduleColorsMeta) = colnames (dat_tmp)
MEsMeta = orderMEs (MEs)
```

```
rownames (MEsMeta) = rownames (dat_tmp)
##create cluster_mapping_file
cluster_mapping_file_m<-
data.frame(New_Name=paste0("M",1:length(MEsMeta)),Description=c("None"))
row.names(cluster_mapping_file_m) <- paste0("M_",colnames(MEsMeta))
cluster_mapping_file_m$label = sapply (rownames (cluster_mapping_file_m),
function (x) paste (cluster_mapping_file_m [x, "New_Name"],
cluster_mapping_file_m [x, "Description"], sep = ": "))
### Determine relevant descriptive statistics of established clusters
### kIN: within-module connectivity, determined by summing connectivity with all
###       other metabolites in the given cluster.
### kME: bicor-correlation between the metabolite profile and module eigenvector;
### both measures of intramodular hub-metabolite status.
kIN <-vector (length = ncol (dat_tmp)); names (kIN) = colnames (dat_tmp)
kME <-vector (length = ncol (dat_tmp)); names (kME) = colnames (dat_tmp)
modules <-vector (length = ncol (dat_tmp)); names (modules) = colnames (dat_tmp)
for (module in names (table (moduleColorsMeta))) {
  all.metabolites = names (dat_tmp)
  inModule = (moduleColorsMeta == module)
  module.metabolites = names (moduleColorsMeta [inModule])
  modules [module.metabolites] = module
  kIN [module.metabolites] = sapply (module.metabolites, function (x) sum (A [x,
module.metabolites]) - 1)
  datKME = signedKME (dat_tmp, MEsMeta, corFnc = corFun_tmp, corOptions =
corOptions_str)
  rownames (datKME) = colnames (dat_tmp)
  kME [module.metabolites] = datKME [module.metabolites, paste ("kME", module,
sep = "")]
}
output = data.frame ("module" = modules, "kME" = kME, "kIN" = kIN,"cluster_name"
= sapply (modules, function (m) cluster_mapping_file_m [paste0 ("M_ME", m),
"New_Name"]))
### Step 6 - Link individual metabolite to phenotype of interest
tmpMat = array (NA, c (ncol (metabolites), 1, 2))
dimnames (tmpMat) [[1]] = colnames (metabolites)
dimnames (tmpMat) [[2]] = c ("age")
dimnames (tmpMat) [[3]] = c ("estimate", "p.value")
### Associating individual metabolites with age
tmpMat [, "age", c ("estimate", "p.value")] =
  t (apply (metabolites, MARGIN = 2, FUN = function (x)
    unlist (cor.test (phenotypes$age, x,
                      method = cor_method, use = "pairwise.complete.obs")[c
("estimate", "p.value")])))
#tmpMat
```

```
### Sort by cluster_name and then decreasing values of kIN
output2 = cbind(tmpMat[, "age", c ("estimate", "p.value")], "p.adjust" = p.adjust (tmpMat [, "age", "p.value"], method = "BH"))
colnames (output2) = paste (rep ("age", 3), colnames (output2), sep = "_")
output3 = cbind (output, output2)
output3 = output3 [with (output3,    order (cluster_name, -kIN)), ]
### Write to file
write.table (output3, file = "individual_metabolites.txt", sep = "\t", col.names = NA, quote = F, row.names = T)
rm (output, output2, output3, tmpMat, dat_tmp)
# step7_合并结果
### Create a joint data frame of metabolite cluster eigengene
### equivalents/effective abundances ('MEsMetLip')
MEsMetLip = MEsMeta [rownames (MEsMeta),]
### Rename module names (columnames) from 'colors' to numbers
colnames (MEsMetLip) = cluster_mapping_file_m [paste0 ("M_", colnames (MEsMeta)), "New_Name"]
### Save MEsMetLip to file, order by module number
write.table (MEsMetLip [ , order (colnames (MEsMetLip))], file = "MEs_metabolites_clusters.txt", sep = "\t", row.names = T, col.names = NA, quote = F)
### Step 8 - Link metabolite clusters to phenotype of interest
### Analogous to Step 6.
### This is a core analysis step generating associations between the
### integrated/clustered –omics data and a clinically interesting phenotype.
cor_age <- list () ### Data structure for storing results of correlation tests under different setups
tmpMat = array (NA, c (ncol (MEsMetLip), 1, 2))
dimnames (tmpMat) [[1]] = names (MEsMetLip)
dimnames (tmpMat) [[2]] = c ("age")
dimnames (tmpMat) [[3]] = c ("estimate", "p.value")
### Associating metabolite clusters with age
tmpMat [, "age", c ("estimate", "p.value")] =
   t (apply (MEsMetLip, MARGIN = 2, FUN = function (x)
      unlist (cor.test (phenotypes$age, x,
                              method = cor_method, use = "pairwise.complete.obs") [c ("estimate", "p.value")])))

cor_age [["metlip"]] <- tmpMat
rm (tmpMat)
### Step 11 - Save phenotype associations
###
### Save the age association of metabolite clusters calculated in Step 8.
###
tmpMat = cor_age [["metlip"]]
```

```
out = cbind (tmpMat [, "age", c("estimate", "p.value")], "p.adjust" = p.adjust (tmpMat
[, "age","p.value"], method = "BH"))
colnames (out) = paste (rep ("age", 3), colnames (out), sep = "_")
write.table (out, file = "age_cluster_metabolites.txt", sep = "\t", row.names = T,
col.names = NA, quote = F)
### Step 12 - Select features with significant differences
###
### Here, combine and integrate those functional, taxonomic and metabolomics
### features which reliably correspond to the host phenotype of interest, then
### later determine their inter-correlations.
final.fdr.cutoffs = 0.1 ### FDR thresholds, change to your likings.
sig_name <- list ()
sig_name [[paste ("fdr", final.fdr.cutoffs, sep = "_")]] [["metlip"]] =
   names (which (p.adjust (tmpMat [ , "age", "p.value"], method = "BH") <
final.fdr.cutoffs))#sig_name
sig_name_metabolites <- row.names(out)[out[,3] < 0.1]
MEsmeta_sig <- MEsMetLip[,sig_name_metabolites]

### bacterial data were calculated in the same way
### -phenotypes
#phenotypes = read.csv (file = "./data/phenotypes.csv", row.names = 1, header = T, sep
= ",")
### -bacteria
# Data conversion
data <- read.csv("bacterial_data.csv",header = T, row.names = 1,sep = ",")
data1 <- data *500000
#head(data1)
data1[data1==0] = 1.0000001
write.csv(data1,"bacteria.csv")
rm(data,data1)
bacteria = read.csv (file = "bacteria.csv", row.names = 1, header = T, sep = ",")
### - cluster_mapping_file_b
#cluster_mapping_file_b = read.csv (file = "./data/cluster_mapping_file_b.csv", header
= T, sep = ",", row.names = 1)
#cluster_mapping_file_b$label =     sapply (rownames (cluster_mapping_file_b),
function (x) paste (cluster_mapping_file_b [x, "New_Name"], cluster_mapping_file_b
[x, "Description"], sep = ": "))
### Step 5 - Identify clusters bacteria
###Settings for WGCNA generally
cor_method= "spearman" ### for association with clinical parameters
corFun_tmp= "bicor"
cluster_method= "average"
corOptions_list= list (use = 'pairwise.complete.obs')
corOptions_str= "use = 'pairwise.complete.obs'"
```

```
BH_pval_asso_cutoff = 0.05
NetworkType= "signed" ### Signed-network (as the PC1 and median profile does not make sense as a summary measure of a cluster with anticorrelated metabolites.)
###Specify data and parameters
dat_tmp = log2 (bacteria) ### work in logarithmic space
### Settings for WGCNA on bacteria measurements
### Once these are established the steps below can be run
RsquareCut_val= 0.89 ### usually ranges 0.80-0.95 but requires inspecting curves
mergingThresh= 0.20 ### Maximum dissimilarity of module eigengenes (i.e. 1-correlation) for merging modules.
minModuleSize= 3 ### minimum number of metabolites constituting a cluster
SoftPower= 13 ### beta-value, main parameter to optimize
### Calculate weighted adjacency matrix
A = adjacency (dat_tmp, power = SoftPower, type = NetworkType, corFnc = corFun_tmp, corOptions = corOptions_str)
colnames (A) = rownames (A) = colnames (dat_tmp)
### Define dissimilarity based on topological overlap
dissTOM = TOMdist (A, TOMType = NetworkType)
colnames (dissTOM) = rownames (dissTOM) = colnames (dat_tmp)
### Hierarchical clustering
metaTree = flashClust (as.dist (dissTOM), method = cluster_method)
### Define modules by cutting branches
moduleLabels1 = cutreeDynamic (dendro = metaTree, distM = dissTOM, method = "hybrid", deepSplit = 4, pamRespectsDendro = T, minClusterSize = minModuleSize)
moduleLabels1 = labels2colors (moduleLabels1)
### Automatically merge highly correlated modules
merge = mergeCloseModules (dat_tmp, moduleLabels1, corFnc = corFun_tmp, corOptions = corOptions_list, cutHeight = mergingThresh)
### Determine resulting merged module colors
moduleLabels2 = merge$colors
### Establish eigengenes of the newly merged modules, used for cluster overall abundances
MEs = merge$newMEs
### Choose final module assignments
moduleColorsMeta = moduleLabels2
names (moduleColorsMeta) = colnames (dat_tmp)
MEsMeta = orderMEs (MEs)
rownames (MEsMeta) = rownames (dat_tmp)
###create cluster_mapping_file_b
cluster_mapping_file_b<-
data.frame(New_Name=paste0("L",1:length(MEsMeta)),Description="None")
row.names(cluster_mapping_file_b) <- paste0("L_",colnames(MEsMeta))
cluster_mapping_file_b$label = sapply (rownames (cluster_mapping_file_b), function (x) paste (cluster_mapping_file_b [x, "New_Name"], cluster_mapping_file_b
```

```
[x, "Description"], sep = ": "))
### Determine relevant descriptive statistics of established clusters
### kIN: within-module connectivity, determined by summing connectivity with all
###           other metabolites in the given cluster.
### kME: bicor-correlation between the metabolite profile and module eigenvector;
### both measures of intramodular hub-metabolite status.
kIN <-vector (length = ncol (dat_tmp)); names (kIN) = colnames (dat_tmp)
kME <-vector (length = ncol (dat_tmp)); names (kME) = colnames (dat_tmp)
modules <-vector (length = ncol (dat_tmp)); names (modules) = colnames (dat_tmp)
for (module in names (table (moduleColorsMeta))) {
   all.metabolites = names (dat_tmp)
   inModule = (moduleColorsMeta == module)
   module.metabolites = names (moduleColorsMeta [inModule])
   modules [module.metabolites] = module
   kIN [module.metabolites] = sapply (module.metabolites, function (x) sum (A [x,
module.metabolites]) - 1)
   datKME = signedKME (dat_tmp, MEsMeta, corFnc = corFun_tmp, corOptions =
corOptions_str)
   rownames (datKME) = colnames (dat_tmp)
   kME [module.metabolites] = datKME [module.metabolites, paste ("kME", module,
sep = "")]
}
output = data.frame ("module" = modules, "kME" = kME, "kIN" = kIN,"cluster_name"
= sapply (modules, function (m) cluster_mapping_file_b [paste0 ("L_ME", m),
"New_Name"]))
### Step 6 - Link individual bacteria to phenotype of interest
tmpMat = array (NA, c (ncol (bacteria), 1, 2))
dimnames (tmpMat) [[1]] = colnames (bacteria)
dimnames (tmpMat) [[2]] = c ("age")
dimnames (tmpMat) [[3]] = c ("estimate", "p.value")
### Associating individual bacteria with age
tmpMat [, "age", c ("estimate", "p.value")] =
   t (apply (bacteria, MARGIN = 2, FUN = function (x)
      unlist (cor.test (phenotypes$age, x,
                               method = cor_method, use = "pairwise.complete.obs")[c
("estimate", "p.value")])))
#head(tmpMat,5)
### Sort by cluster_name and then decreasing values of kIN
output2 = cbind(tmpMat[, "age", c ("estimate", "p.value")], "p.adjust" = p.adjust
(tmpMat [, "age", "p.value"], method = "BH"))
colnames (output2) = paste (rep ("age", 3), colnames (output2), sep = "_")
output3 = cbind (output, output2)
output3 = output3 [with (output3,    order (cluster_name, -kIN)), ]
### Write to file
```

```
write.table (output3, file = "individual_bacteria.txt", sep = "\t", col.names = NA, quote = F, row.names = T)
rm (output, output2, output3, tmpMat, dat_tmp)
# step7_combine the results
### Create a joint data frame of bacteria cluster eigengene
### equivalents/effective abundances ('MEsMetLip')
MEsMetLip = MEsMeta [rownames (MEsMeta),]
### Rename module names (columnames) from 'colors' to numbers
colnames (MEsMetLip) = cluster_mapping_file_b [paste0 ("L_", colnames (MEsMeta)), "New_Name"]
### Save MEsMetLip to file, order by module number
write.table (MEsMetLip [ , order (colnames (MEsMetLip))], file = "MEs_bacteria_clusters.txt", sep = "\t", row.names = T, col.names = NA, quote = F)
### Step 8 - Link bacteria clusters to phenotype of interest
### Analogous to Step 6.
### This is a core analysis step generating associations between the
### integrated/clustered –omics data and a clinically interesting phenotype.
cor_age <- list () ### Data structure for storing results of correlation tests under different setups
tmpMat = array (NA, c (ncol (MEsMetLip), 1, 2))
dimnames (tmpMat) [[1]] = names (MEsMetLip)
dimnames (tmpMat) [[2]] = c ("age")
dimnames (tmpMat) [[3]] = c ("estimate", "p.value")
### Associating bacteria clusters with age
tmpMat [, "age", c ("estimate", "p.value")] =
   t (apply (MEsMetLip, MARGIN = 2, FUN = function (x)
      unlist (cor.test (phenotypes$age, x,
                              method = cor_method, use = "pairwise.complete.obs") [c ("estimate", "p.value")])))
cor_age [["metlip"]] <- tmpMat
rm (tmpMat)
### Step 11 - Save phenotype associations
###
### Save the age association of bacteria clusters calculated in Step 8.
###
tmpMat = cor_age [["metlip"]]
out = cbind (tmpMat [, "age", c("estimate", "p.value")], "p.adjust" = p.adjust (tmpMat [, "age","p.value"], method = "BH"))
colnames (out) = paste (rep ("age", 3), colnames (out), sep = "_")
write.table (out, file = "age_cluster_bacteria.txt", sep = "\t", row.names = T, col.names = NA, quote = F)
### Step 12 - Select features with significant differences
###
### Here, combine and integrate those functional, taxonomic and metabolomics
```

```
### features which reliably correspond to the host phenotype of interest, then
### later determine their inter-correlations.
final.fdr.cutoffs = 0.1 ### FDR thresholds, change to your likings.
sig_name <- list ()
sig_name [[paste ("fdr", final.fdr.cutoffs, sep = "_")]] [["metlip"]] =
  names (which (p.adjust (tmpMat [ , "age", "p.value"], method = "BH") < final.fdr.cutoffs))

#sig_name
age_cluster_bacteria <- read.table("age_cluster_bacteria.txt",sep = "\t", header = T, row.names = 1)
sig_name_bacteria <- row.names(age_cluster_bacteria)[age_cluster_bacteria$age_p.adjust < 0.1]
MEsbac <- read.table("MEs_bacteria_clusters.txt",sep = "\t", header = T, row.names = 1)
MEsbac_sig <- MEsbac[,sig_name_bacteria]
### Step 13 - Correlate metabolite clusters to bacteria clusters
###
### The set of metabolite clusters associated with the phenotype of interest
### should next be tested for association with the set of bacteria
### clusters likewise so associated (identified in Step 12).
###
### In this step, the correlations between each metabolite cluster and
### microbiota cluster are determined.
###
# Metabolites clusters with significant performance were extracted
# sig_name_metabolites <- row.names(out)[out[,3] < 0.1]
# MEsmeta_sig <- MEsMetLip[,sig_name_metabolites]
# # Read the intestinal bacteria cluster, extract the rows with age_P.adjust < 0.1, read the data matrix of intestinal bacteria after dimensionality reduction, and extract the rows with significance#
# age_cluster_bacteria <- read.table("./result/age_cluster_bacteria.txt",sep = "\t", header = T, row.names = 1)
# sig_name_bacteria <- row.names(age_cluster_bacteria)[age_cluster_bacteria$age_p.adjust < 0.1]
# MEsbac <- read.table("./result/MEs_bacteria_clusters.txt",sep = "\t", header = T, row.names = 1)
# MEsbac_sig <- MEsbac[,sig_name_bacteria]
# metabolite cluster and microbiota cluster
bac_MEtlip_cor = matrix (NA, nrow = ncol (MEsbac_sig), ncol = ncol (MEsmeta_sig))
dim(bac_MEtlip_cor)
rownames (bac_MEtlip_cor) = colnames (MEsbac_sig)
colnames (bac_MEtlip_cor) = colnames (MEsmeta_sig)
bac_MEtlip_p = bac_MEtlip_cor
```

```
bac_MEtlip_p.adjust = bac_MEtlip_cor
for (m in colnames (MEsmeta_sig)) {
   bac_MEtlip_cor [ , m] = apply (MEsbac_sig, MARGIN = 2, FUN = function (x)
      cor.test (x, MEsmeta_sig[,m], method = cor_method, use =
"pairwise.complete.obs")$estimate)
   bac_MEtlip_p[,m] = apply (MEsbac_sig, MARGIN = 2, FUN = function (x)
      cor.test (x, MEsmeta_sig[,m], method = cor_method, use =
"pairwise.complete.obs")$p.value)}
bac_MEtlip_p.adjust = apply(bac_MEtlip_p, 2, FUN = function(x)
   p.adjust(x,method = "BH"))
write.csv(bac_MEtlip_cor,"bac_MEtlip_cor.csv")
write.csv(bac_MEtlip_p, "bac_MEtlip_p.csv")
write.csv(bac_MEtlip_p.adjust, "bac_MEtlip_p.adjust.csv")
```

#Sankey Diagram

```
library(ggalluvial)
library("reshape2")
CAG <- read.csv("plot_CAG_M_fdr.csv")
cytokine <- read.csv("plot_M_cytokine_fdr.csv")
M<- merge(CAG,cytokine,by="M")
ggplot(M,
         aes(y = r1, axis1 = CAG, axis2 = M)) +
   scale_x_discrete(limits = c("CAG", "M"), expand = c(.1, .05)) +
   geom_alluvium(aes(fill = r1)) +
   geom_stratum() +
   geom_text(stat = "stratum", aes(label = after_stat(stratum))) +
   theme_minimal()
ggplot(M,
         aes(y = r1, axis1 = M, axis2 = cytokine)) +
   scale_x_discrete(limits = c("M", "cytokine"), expand = c(.1, .05)) +
   geom_alluvium(aes(fill = r2)) +
   geom_stratum() +
   geom_text(stat = "stratum", aes(label = after_stat(stratum))) +
   theme_minimal()
```

###mantel test

```
library(dplyr)
#> Warning: package 'dplyr' was built under R version 3.6.2
data("varechem", package = "vegan")
data("varespec", package = "vegan")

mantel <- mantel_test(varespec, varechem,
```

```
                              spec.select = list(Spec01 = 1:7,
                                                 Spec02 = 8:18,
                                                 Spec03 = 19:37,
                                                 Spec04 = 38:44)) %>%
  mutate(rd = cut(r, breaks = c(-Inf, 0.2, 0.4, Inf),
                  labels = c("< 0.2", "0.2 - 0.4", ">= 0.4")),
         pd = cut(p.value, breaks = c(-Inf, 0.01, 0.05, Inf),
                  labels = c("< 0.01", "0.01 - 0.05", ">= 0.05")))

mantel_test(varespec, varechem,
            spec.select = list(spec01 = 1:5, spec02 = 6:12))
mantel_test(varespec, varechem,
            spec.select = list(spec01 = 1:5, spec02 = 6:12),
            env.select = list(env01 = 1:5, env02 = 6:10, env03 = 11:14))
mantel_test(varespec, varechem,
            env.ctrl = 11:14,
            spec.select = list(spec01 = 1:5, spec02 = 6:12),
            env.select = list(env01 = 1:5, env02 = 6:10))
set.seed(20191224)
sam_grp <- sample(paste0("sample", 1:3), 24, replace = TRUE)
mantel_test(varespec, varechem, group = sam_grp)

quickcor(varechem, type = "upper") +
  geom_square() +
  anno_link(aes(colour = pd, size = rd), data = mantel) +
  scale_size_manual(values = c(0.5, 1, 2)) +
  scale_colour_manual(values = c("#D95F02", "#1B9E77", "#A2A2A288")) +
  guides(size = guide_legend(title = "Mantel's r",
                             override.aes = list(colour = "grey35"),
                             order = 2),
         colour = guide_legend(title = "Mantel's p",
                               override.aes = list(size = 3),
                               order = 1),
         fill = guide_colorbar(title = "Pearson's r", order = 3))###end##
```

###mediation analysis: microbial impacts on Immune inflammation through metabolites

```
library(lme4)
library(mediation)
t2<-read.csv("data.csv", header = T, row.names = 1)
model.m=lm(meta ~ microbe+age+sex,t2)
model.y=lm(cytokine ~ microbe+meta+age+sex,t2)
summary=summary(mediate(model.m ,model.y,treat = "microbe", mediator =
```

```
"meta",boot = F,sims = 1000))
plot(summary)
summary(summary)
```

###Random forest model and ROC

```
#function
rfcv1 <-function (trainx, trainy, cv.fold = 5, scale = "log", step = 0.5, mtry = function(p)
max(1, floor(sqrt(p))), recursive = FALSE, ...)
{
  classRF <- is.factor(trainy)
  n <- nrow(trainx)
  p <- ncol(trainx)
  if (scale == "log") {
    k <- floor(log(p, base = 1/step))
    n.var <- round(p * step^(0:(k - 1)))
    same <- diff(n.var) == 0
    if (any(same))
      n.var <- n.var[-which(same)]
    if (!1 %in% n.var)
      n.var <- c(n.var, 1)
  }
  else {
    n.var <- seq(from = p, to = 1, by = step)
  }
  k <- length(n.var)
  cv.pred <- vector(k, mode = "list")
  for (i in 1:k) cv.pred[[i]] <- rep(0,length(trainy))
  if (classRF) {
    f <- trainy
  }
  else {
    f <- factor(rep(1:5, length = length(trainy))[order(order(trainy))])
  }
  nlvl <- table(f)
  idx <- numeric(n)
  for (i in 1:length(nlvl)) {
    idx[which(f == levels(f)[i])] <- sample(rep(1:cv.fold,
                                                 length = nlvl[i]))
  }
  res=list()
  for (i in 1:cv.fold) {
    all.rf <- randomForest(trainx[idx != i, , drop = FALSE],
                           trainy[idx != i],importance = TRUE)
```

```
    aa = predict(all.rf,trainx[idx == i, , drop = FALSE],type="prob")
    cv.pred[[1]][idx == i] <- as.numeric(aa[,2])
    impvar <- (1:p)[order(all.rf$importance[, 3], decreasing = TRUE)]
    res[[i]]=impvar
    for (j in 2:k) {
      imp.idx <- impvar[1:n.var[j]]
      sub.rf <- randomForest(trainx[idx != i, imp.idx,
                                    drop = FALSE], trainy[idx != i]
      )
      bb <- predict(sub.rf,trainx[idx ==i,imp.idx, drop = FALSE],type="prob")
      cv.pred[[j]][idx == i] <- as.numeric(bb[,2])
      if (recursive) {
        impvar <- (1:length(imp.idx))[order(sub.rf$importance[,
                                                               3],
decreasing = TRUE)]
      }
      NULL
    }
    NULL}
  if (classRF) {
    error.cv <- sapply(cv.pred, function(x) mean(factor(ifelse(x>0.5,1,0))!=trainy))
  }
  else{
    error.cv <- sapply(cv.pred, function(x) mean((trainy -
                                                    x)^2))
  }
  names(error.cv) <- names(cv.pred) <- n.var
  list(n.var = n.var, error.cv = error.cv, predicted = cv.pred,res=res)
}
##Read the data
set.seed(1234)
library(randomForest)
library(pROC)
data <- read.csv("all_bac_meta.csv", header = T)
index <- sample(2,nrow(data),replace = TRUE,prob=c(0.7,0.3))
train <- data[index==1,]
test <- data[index==2,]
train$group<-as.factor(train$group)
######5*10_crossvalidation####
##source("ramdomforest.crossvalidation.r")
result <- replicate(5, rfcv1(train[,-ncol(train)], train$group, cv.fold=10,step=0.9),
simplify=FALSE)
error.cv <- sapply(result, "[[", "error.cv")
matplot(result[[1]]$n.var, cbind(rowMeans(error.cv), error.cv), type="l", lwd=c(2,
```

```
rep(1, ncol(error.cv))), col=1, lty=1, log="x", testlab="Number of variables", ylab="CV
Error")
abline(v=5,col="pink",lwd=2)
error.cv.cbm<-cbind(rowMeans(error.cv), error.cv)
cutoff<-min (error.cv.cbm[,1])+sd(error.cv.cbm[,1])
error.cv.cbm[error.cv.cbm[,1]<cutoff,]
#####pick 10 marker by corossvalidation#######
k=1
b <- matrix(0,ncol=184,nrow=50)
for(i in 1:5){
  for(j in 1:10){
    b[k,]<-result[[i]]$res[[j]]
    k=k+1 } }
mlg.list<-b[,1:10]
list<-c()
k=1
for(i in 1:10){
  for(j in 1:50){
    list[k]<-mlg.list[j,i]
    k=k+1 } }
mlg.sort<-as.matrix(table(list))
mlg.sort<-mlg.sort[rev(order(mlg.sort[,1])),]
pick<- as.numeric(names(head(mlg.sort,5)))
tmp=train[,-ncol(train)]
mlg.pick<-colnames(tmp)[pick]
write.table(mlg.pick,"crossvalidation.ttestt", sep="\t",quote=F)
###train.set
#train1 <- train[,c(pick,127)]
set.seed(1234)
train1 <-data.frame(train)
train1.rf <- randomForest(group ~ 4,8 Dimethylnonanoyl carnitine
                          + Cholic acid
                          + PE(22:6(4Z,7Z,10Z,13Z,16Z,19Z)/P-18:1(11Z))
                          + Prostaglandin A2
                          + 3-Hydroxycapric acid
                          + PC(P-18:1(11Z)/22:5(4Z,7Z,10Z,13Z,16Z))
                          + PE(20:4(8Z,11Z,14Z,17Z)/P-18:1(11Z))
                          , data =train, importance = TRUE)
varImpPlot(train1.rf)
train1.pre <- predict(train1.rf,type="prob")
p.train<-train1.pre[,2]
boxplot(p.train~train$group,col=c(3,4),main="Probability of schizophrenia")
write.table(p.train,"predict.in.train.ttestt", sep="\t",quote=F)
########ROC in train######
```

```
library(pROC)
roc(train$group,p.train)
####################
roc1 <- roc(train$group, p.train, percent=TRUE, partial.auc.correct=TRUE, ci=TRUE, boot.n=100, ci.alpha=0.9, stratified=FALSE, plot=F, auc.polygon=TRUE, matest.auc.polygon=TRUE, grid=TRUE )
######################
roc1 <- roc(train$group, p.train, ci=TRUE, boot.n=100, ci.alpha=0.9, stratified=FALSE, plot=TRUE, percent=roc1$percent,col=2)
sens.ci <- ci.se(roc1, specificities=seq(0, 100, 5))
plot(sens.ci, type="shape", col=rgb(0,1,0,alpha=0.2))
plot(sens.ci, type="bars")
plot(roc1,col=2,add=T)
legend("bottomright",c(paste("AUC=",round(roc1$ci[2],2),"%"), paste("95% CI:",round(roc1$ci[1],2),"%-",round(roc1$ci[3],2),"%")))
##end##
##########test.set###########
set.seed(999)
predict(train1.rf, test)
set.seed(999)
test1.pre<-predict(train1.rf, test,type="prob")
p.test<-test1.pre[,2]
boxplot(p.test~test$group,col=c(3,4),main="Probability of schizophrenia")
write.table(p.test,"predict.in.test.ttestt", sep="\t",quote=F)
########ROC in test######
roc(test$group,p.test)
####################
roc1 <- roc(test$group, p.test, percent=TRUE, partial.auc.correct=TRUE, ci=TRUE, boot.n=100, ci.alpha=0.9, stratified=FALSE, plot=F, auc.polygon=TRUE, matest.auc.polygon=TRUE, grid=TRUE )
######################
roc1 <- roc(test$group, p.test, ci=TRUE, boot.n=100, ci.alpha=0.9, stratified=FALSE, plot=TRUE, percent=roc1$percent,col=2)
sens.ci <- ci.se(roc1, specificities=seq(0, 100, 5))
plot(sens.ci, type="shape", col=rgb(0,1,0,alpha=0.2))
plot(sens.ci, type="bars")
plot(roc1,col=2,add=T)
legend("bottomright",c(paste("AUC=",round(roc1$ci[2],2),"%"), paste("95% CI:",round(roc1$ci[1],2),"%-",round(roc1$ci[3],2),"%")))
##end##
```
